# Combining Cationic Liposomal Delivery with MPL-TDM for Cysteine Protease Cocktail Vaccination against *Leishmania donovani* : Evidence for Antigen Synergy and Protection

**DOI:** 10.1371/journal.pntd.0003091

**Published:** 2014-08-21

**Authors:** Amrita Das, Nahid Ali

**Affiliations:** Infectious Diseases and Immunology Division, Indian Institute of Chemical Biology, Jadavpur, Kolkata, India; Institut Pasteur de Tunis, Tunisia

## Abstract

**Background:**

With the paucity of new drugs and HIV co-infection, vaccination remains an unmet research priority to combat visceral leishmaniasis (VL) requiring strong cellular immunity. Protein vaccination often suffers from low immunogenicity and poor generation of memory T cells for long-lasting protection. Cysteine proteases (CPs) are immunogenic proteins and key mediators of cellular functions in *Leishmania*. Here, we evaluated the vaccine efficacies of CPs against VL, using cationic liposomes with Toll like receptor agonists for stimulating host immunity against *L. donovani* in a hamster model.

**Methodology/Principal Findings:**

Recombinant CPs type I (*cpb*), II (*cpa*) and III (*cpc*) of *L. donovani* were tested singly and in combination as a triple antigen cocktail for antileishmanial vaccination in hamsters. We found the antigens to be highly immunoreactive and persistent anti-CPA, anti-CPB and anti-CPC antibodies were detected in VL patients even after cure. The liposome-entrapped CPs with monophosphoryl lipid A-Trehalose dicorynomycolate (MPL-TDM) induced significantly high nitric oxide (up to 4 fold higher than controls) mediated antileishmanial activity in vitro, and resulted in strong in vivo protection. Among the three CPs, CPC emerged as the most potent vaccine candidate in combating the disease. Interestingly, a synergistic increase in protection was observed with liposomal CPA, CPB and CPC antigenic cocktail which reduced the organ parasite burden by 10^13^–10^16^ folds, and increased the disease-free survival of >80% animals at least up to 6 months post infection. Robust secretion of IFN-γ and IL-12, along with concomitant downregulation of Th2 cytokines, was observed in cocktail vaccinates, even after 3 months post infection.

**Conclusion/Significance:**

The present study is the first report of a comparative efficacy of leishmanial CPs and their cocktail using liposomal formulation with MPL-TDM against *L. donovani*. The level of protection attained has not been reported for any other subcutaneous single or polyprotein vaccination against VL.

## Author Summary

Conventional chemotherapy of visceral leishmaniasis (VL) typically relies on pentavalent antimonials that suffer from extensive drug resistance in India. Development of preventive vaccination is undoubtedly a better alternative to completely eradicate the disease. With this in mind, we chose to target parasite cysteine proteases (CPs), with immense biological importance, as potential vaccine candidates against *Leishmania donovani*. Here, we describe the superior efficacy of an antigenic cocktail of type I, II and III CPs entrapped in cationic liposomes with Toll like receptor (TLR) agonists: monophosphoryl lipid A- Trehalose dicorynomycolate (MPL-TDM), against *L. donovani* in a hamster model. The three CPs acted synergistically in the cocktail to induce almost complete protection against *Leishmania*. The protection is chiefly mediated through upregulation of protective cytokines like interferon-gamma (IFN-γ), interleukin-12 (IL-12), IL-2, and tumour necrosis factor (TNF-α), with concomitant down-regulation of disease promoting cytokines, like transforming growth factor–beta (TGF-β), IL-10 and IL-4. The antigens were also compared singly for their protective potential. Interestingly, type III (CPC) CP emerged as the most potent antigenic component of the cocktail inducing better protection than type I and II. Hence, the cysteine proteases of *Leishmania* form an attractive group of vaccine candidates for future studies in human VL.

## Introduction

Visceral leishmaniasis (VL) caused by *Leishmania donovani* is a fatal disease with an estimated 360,000 new cases all over the world with almost 10% annual case fatality in the Indian subcontinent alone [1]. It is a neglected tropical disease inevitably associated with poverty and immunosuppression. High toxicity of available drugs (amphotericin B, miltefosine and paromomycin), HIV co-infection, and resistant parasites pose a global threat against leishmaniases. Despite recent advances in pharmaceutics and molecular immunology, there is no licensed vaccine available against the disease till date [2]. Encapsulation of antigens within nanocarriers promises stable and customized vaccine delivery to related immune cells against various intracellular pathogens including *Leishmania*. Although incompletely understood, the lack of clinical efficacy for peptide-based vaccines may be related to several factors such as poor immunogenicity of the subunit antigens, inappropriate functional polarity of T cells and *Leishmania* induced immunosuppression [2], [3]. Thus, there remains considerable scope for improvement of antileishmanial vaccine design to maximize the chances of clinical benefit. Outcome of prophylactic vaccination largely depends on the choice of right immunopotentiating adjuvants and/or delivery systems coupled to right antigen(s). Cationic liposomes protect the labile antigens from lysosomal degradation and take the advantage of electrostatic interactions with the cells' negative charge which makes them a natural target for antigen presenting cells (APCs), crucial for immune stimulation [4], [5]. Monophosphoryl lipid A (MPLA) is a Toll-like receptor 4 (TLR4) agonist with more than 100,000 human doses safely administered as a part of licensed hepatitis B and Human papillomavirus vaccines [6]. Mycobacterial glycolipid trehalose-6,6′-dimycolate (TDM; cord factor) is a potent immunostimulant known for its macrophage activation properties and induction of proinflammatory cytokines, and anti-tumor activity [7]. Recently, TDM has been shown to act via macrophage receptor with collagenous structure (MARCO), TLR2, CD14 and also macrophage-inducible C-type lectin (Mincle) receptors to exert its immunomodifying effects [8], [9]. When used together, both the adjuvants i.e. MPL and TDM non-specifically activate the immune system, allowing a better response to the associated immunogen [10]. Recently, we have developed a cationic liposome and MPL-TDM (monophosphoryl lipid-trehalose dicorynomycolate) delivery platform that is suitable for subcutaneous delivery of leishmanial antigens in mice model [11].

Compared to an array of antigens that have been tested, very few are sufficiently promising to be carried out to Phase I clinical trials or advanced preclinical work against VL [12]. Lysosomal cysteine proteases (CP) of *Leishmania*, including cysteine proteases type I (CPB), II (CPA) and III (CPC), are conserved and functionally important proteolytic enzymes regulating cell cycle and host-parasite interactions that have been found to be immunogenic in mice. Further, CPs are established virulence factors involved in parasitic survival, autophagy and metacyclogenesis essential for onset of the disease and are being increasingly exploited as serodiagnostic markers, drug targets and vaccine candidates against *Leishmania* [13], [14]. Protein and DNA vaccine attempts using CPA, CPB and CPC in different adjuvants have been reported against *L. major* [15], [16] and *L. infantum* [17] in mice and canine models [18]. However, these research efforts have been largely restricted to cutaneous and zoonotic VL with variable success rate but have never been investigated against *L. donovani*, the causative agent of kala-azar. Although mouse serves as a good animal model for dissecting the protective immune responses with the available immunological reagents, murine infection is usually self-curing and differs from human VL. In contrast, hamsters closely mimic clinical symptoms of human VL characterized by severe immunosuppression (Th2 response) and develop progressive fatal infection when challenged with *L. donovani*. Higher innate susceptibility of this model compared to mice makes them a better choice for initial vaccine trial against VL to ensure its success in human [19]. Hence we selected Syrian golden hamsters to assess the protective potential of leishmanial CPs. Our aim in this study was therefore towards a comparative evaluation of prophylactic potential of CPA, CPB and CPC, individually, as well as a multi-antigen cocktail vaccine, maximizing the exposed antigenic epitopes to protect against VL. Evidence is presented here for the first time on the efficacy of highly potent liposomal formulations of recombinant CPA, CPB and CPC along with MPL-TDM adjuvant, in a hamster model against *L. donovani*, with an aim towards its further development for human vaccination.

## Materials and Methods

### Ethics statement

The present study was approved by the Ethical Committee on Human Subjects at the Indian Institute of Chemical Biology and Ethical Committee, Calcutta School of Tropical Medicine, Kolkata. Written informed consent was obtained from each patient and healthy donors enrolled in the study and the patient/parent was informed that he/she was free to voluntarily withdraw from the study at any time. Written consent was obtained from parent or guardian in case of minors. The physicians explained the nature of the investigation and the risks involved to each patient (or parent or guardian in case of minors), prior to recruitment. A copy of the patient consent form was submitted to the Ethical Committee.

The animal experiments were approved by the Animal Ethical Committee (147/1999/CPSCEA) of the institute, according to the National Regulatory Guidelines issued by the Committee for the Purpose of Control and Supervision on Experimental Animals (CPCSEA), under the Division of Animal Welfare, Ministry of Environment and Forest, Government of India.

### Animals and parasites

Studies were performed with 4–6 weeks old Syrian golden hamsters (*Mesocricetus auratus*) reared in pathogen-free animal care facility of the Indian Institute of Chemical Biology. A strain of *L. donovani* (MHOM/IN/83/AG83) originally isolated from an Indian kala-azar patient was maintained by serial passage in Syrian golden hamsters as described earlier [20]. Parasites from stationary-phase culture were sub-cultured to maintain an average density of 2×10^6^ cells/ml.

### Cloning, expression and purification of *L. donovani* cysteine proteases *a*, *b* and *c*

Plasmids containing full-length *cpa* (GenBank accession number KF018070), *cpb* (GenBank accession number KC609324) and *cpc* (GenBank accession number JX968801.1) from *L. donovani* (pET28a-*cpa*, pET28a-*cpb* and pET28a-*cpc*) were cloned, expressed and purified from inclusion bodies using sarkosyl (Sigma-aldrich, St. Louis, MO) [21]. Genomic DNA isolated from *L. donovani* promastigotes was subjected to polymerase chain reaction (PCR) with sets of gene specific primers corresponding to *cpa*, *cpb* and *cpc* genes based on *Leishmania major* and *Leishmania infantum* gene sequences (Table S1, supporting information). PCR conditions for rCPA and rCPB were one cycle of 5 min at 94°C, 35 cycles of 1 min at 94°C, 1 min at 59°C, and 1 min 10 s at 72°C, followed by a final cycle of 7 min at 72°C. PCR conditions for rCPC were one cycle of 5 min at 94°C, 35 cycles of 1 min at 94°C, 1 min 20 s at 58°C, and 1 min 10 s at 72°C, followed by a final cycle of 7 min at 72°C. The PCR amplified fragments were separately cloned into NdeI/BamHI site of bacterial expression vector pET28a (Novagen, Madison, USA). All the restriction enzymes were from Roche Diagnostics, Mannheim, Germany. Screening for recombinant clones was performed by growing randomly selected colonies overnight at 37°C in 5 ml of Luria-Bertani (LB) broth (with 35 µg/µl kanamycin). Plasmid DNA were extracted from bacterial cell pellets using the QIAprep Spin Miniprep Kit (Qiagen, Valencia, USA), following the manufacturer's instructions. For clone confirmation, approximately 1 µg plasmid DNA from an individual miniprep was double digested with the appropriate restriction enzymes (NdeI and HindIII) and the digest loaded onto a 1% agarose gel, in parallel with the molecular weight marker: 1 kb DNA ladder (Fermentas, USA). Positive clones were selected on the basis of the size of the insert and confirmed by DNA sequencing (ABI Prism, Model 377; Applied Biosystems). *Escherichia coli* BL21 (DE3) transformed with each of pET28a-*cpa*, pET28a-*cpb* and pET28a-*cpc* constructs were grown in 500 ml of culture medium at 37°C until logarithmic phase OD_600_ reached 0.6. Protein production was induced by adding isopropyl β-d-thiogalactoside (IPTG) to a final concentration of 0.5 mM, and incubating for an additional 4 h at 30°C. The culture was then harvested by centrifugation at 6,000×*g*, for 6 min, at 4°C, and the cell pellet was resuspended in 6 ml of resuspension buffer [25 mM Tris-HCl, 500 mM NaCl, and 1 mg/ml of Lysozyme (Roche), pH 8.0]. The cell lysate was sonicated on ice for 5 min with 1 min pulse and 1 min interval between pulses using an ultrasonicator (Misonix, Farmingdale, NY, USA). The pellet containing inclusion bodies was solubilised with solublization buffer [50 mM CAPS {3-(Cyclohexylamino)-1-propanesulfonic acid} buffer (pH 11.0), 300 mM NaCl, and 0.5% sarkosyl], kept at room temperature for 30 min and finally centrifuged at 12,000×*g* for 30 min at 4°C. The supernatant containing solubilised proteins were loaded separately onto Ni^2+^-nitrilotriacetic acid-agarose (Ni-NTA) column (Qiagen) and purified under denaturing conditions [16]. The Ni-NTA column was pre equilibriated with equilibriation buffer [50 mM CAPS buffer (pH 11.0), 150 mM NaCl, 0.5% sarkosyl and 10 mM imidazole]. The column was washed with wash buffer [50 mM CAPS buffer (pH 11.0), 150 mM NaCl, 0.5% sarkosyl and 20 mM imidazole] and eluted with elution buffer [50 mM CAPS buffer (pH 11.0), 150 mM NaCl, 0.5% sarkosyl and 300 mM imidazole]. Bacterial endotoxins were largely removed by three consecutive washing of the protein bound Ni-NTA agarose beads with wash buffer containing 0.5% and 0.1% (v/v) Triton X-114 (Sigma-Aldrich) [22]. This was followed by washing the beads without the detergent (in 20 volumes of wash buffer without Triton X-114) prior to elution. The endotoxin level in each of the recombinant proteins was determined using the chromogenic Limulus Amebocyte Lysate (LAL) assay kit (QCL-1000; Lonza) according to the manufacturer's recommendations. This step was repeated as required to maintain the endotoxin content of the purified recombinant proteins as <0.2 endotoxin units/µg. To refold, the purified materials were diluted 2 fold in dilution buffer containing 50 mM CAPS buffer (pH 11.0), 150 mM NaCl and 300 mM imidazole, and then dialyzed against 25 mM Tris-HCl, 250 mM NaCl, pH 8.0 and finally in 0.02 M PBS for 6 h at 4°C. Protein concentrations were determined using Lowry's method [23]. Purity and homogeneity of the purified proteins were checked on10% sodium dodecyl sulfate (SDS)-PAGE, and the gel was subsequently stained with Coomassie Brilliant Blue R-250 (Bio-Rad Laboratories).

ClustalW DNA alignment was performed using the ClustalW program of the European Bioinformatics Institute (http://www.genome.jp/tools-bin/clustalw) and multiple alignment was used to view the ClustalW alignment [24]. Cysteine protease gene sequences of different *Leishmania* species were derived from the GenBank.

### Detection of anti-CPA, anti-CPB and anti-CPC antibodies in VL patients

Blood plasma samples were obtained from five patients confirmed with active VL, admitted between 2009 and 2012 to the School of Tropical Medicine (Kolkata, India) mainly from endemic regions of Bihar and West Bengal. Five recovered cases of liposomal amphotericin B -treated patients and five normal individuals negative for the recombinant K39 strip test from a non-endemic area were included as cured and healthy controls respectively. HIV-positive individuals with VL and pregnant women were excluded from study. Heparinized, longitudinal blood samples were collected from the patients (both with active VL and cured) as well as healthy individuals from which about 1 ml of plasma was used for the study. Plasma was obtained from the upper layer of the gradient following centrifugation and stored at −20°C until use. Purified CPs (rCPA, rCPB and rCPC) (5 µg) were subjected to 10% SDS-polyacrylamide gel electrophoresis [25], transferred (Mini-Protean II, Bio-Rad) to nitrocellulose membrane at 85 V for 1 h and incubated with human plasma (1∶1000) from patients with active VL, cured and healthy individuals. The membranes were washed and probed with horseradish peroxidase (HRP)-conjugated goat anti-human IgG (1∶4000) (Southern Biotech) as secondary antibody. The present study was approved by the Ethical Committee on Human Subjects at the Indian Institute of Chemical Biology.

### Preparation of DSPC-bearing cationic liposomes and entrapment of recombinant proteins

Distearoyl phosphatidylcholine (DSPC), cholesterol (Sigma-aldrich) and stearylamine (Fluka, Buchs, Switzerland) at a molar ratio of 7∶2∶2 were dissolved in chloroform followed by evaporating the organic solvents to form a thin film as described earlier [26]. Empty and antigen entrapped liposomes were prepared by dispersion of lipid film in either 1 ml of 0.02 M phosphate buffered saline (PBS) alone or containing 500 µg/ml of recombinant CPA (rCPA), CPB (rCPB) or CPC (rCPC). The mixture was then vortexed and the suspension was sonicated for 30 s by an ultrasound probe sonicator (Misonix, New York, USA) thrice with 1 min gap in between at 4°C. It was then kept on ice for 2 h to stabilize the formed liposomes. Vesicles with entrapped antigen were separated from excess free antigens by three successive washings in PBS with ultracentrifugation at 105,000× *g* for 1 h at 4°C.The protein entrapped in liposome was estimated using BSA as the standard, in presence of 0.8% SDS and appropriate blanks [23]. Protein integrity after liposomal encapsulation was also evaluated by 10% SDS-PAGE followed by Coomassie staining.

For fluorescent liposomes, the lipid film was made with DSPC, cholesterol and stearylamine at a molar ratio of 7∶2∶2 along with 0.1 mg/ml of rhodamine 123 (Rh123) (Sigma Life Sciences) as a lipophilic marker. The dry lipid film was dispersed in 0.02 M PBS and the excess free dye was separated from labeled liposomes by three successive washings as described above.

### Characterization of liposomes by atomic forced microscopy

For atomic forced microscopy (AFM) imaging of liposomal samples, 10 µl of the samples were deposited onto freshly cleaved muscovite Ruby mica sheets (ASTM V1 Grade Ruby Mica from MICAFAB) for 15–20 minutes. Mica sheets are basically negatively charged so samples bind strongly on the mica surface. After 15 min, the samples were dried by using a vacuum dryer. Sometimes the samples were gently washed with 0.5 ml Milli-Q water to remove molecules that were not firmly attached to the mica and the samples were dried as mentioned above. Acoustic alternative current mode AFM was performed using a Pico plus 5500 ILM AFM (Agilent Technologies, USA) with a piezoscanner maximum range of 9 µm. Micro fabricated silicon cantilevers of 225 µm in length with a nominal spring force constant of 21–98 N/m were used from Nano sensors, USA. Cantilever oscillation frequency at 150–300 kHz was tuned into resonance frequency. The images (512 by 512 pixels) were captured with a scan size between 0.5 and 2 µm at a scan speed rate of 0.5lines/S. Images were flattened using Pico view1.4 version software (Agilent Technologies). Image processing and analyzation was done through Pico Image Advanced version software (Agilent Technologies).

### Flowcytometry

The mechanism of cellular uptake of liposomes was quantified by fluorescence activated cell sorting (FACS) using various biochemical inhibitors. Macrophages collected by peritoneal lavage of healthy, adult Syrian golden hamsters were incubated overnight in a 24-well flat bottom plate at a density of 1×10^6^ cells/well in RPMI 1640/10% fetal bovine serum (FBS). Cells were incubated in serum free RPMI 1640 with various biochemical inhibitors (all from Sigma-Aldrich): 500 µM amiloride, 5 µg/ml chlorpromazine, 50 µg/ml cytochalasin D for 30 min and 10 µM colchicine for 2 h to block macropinocytosis, non-clathrin, non-caveolae dependent endocytosis, actin and microtubules mediated endocytosis, respectively [27]–[29]. Cells were subsequently treated with rhodamine 123 (Rh123)-labelled cationic liposomes (final concentration 0.2 mg lipid/ml with respect to DSPC) for 1 h in the presence of the inhibitors. Cells incubated with labelled liposomes without prior treatment with inhibitors served as positive controls. Cells were detached using trypsin/EDTA, washed thrice with ice-cold 0.02 M PBS containing 0.5% FBS, and centrifuged at 2000 rpm g for 10 minutes to remove the liposomes adhered to the cell surface and analysed by flow cytometry using a FACS LSR Fortessa (Becton Dickinson) and FACSDiva software (BD Biosciences).

### Immunization and challenge infection

All vaccines were formulated with cationic liposomes as described previously. Six groups (35 hamsters/group for controls and 25 hamsters/group for antigens) were immunized subcutaneously between scapulae at the back, two times at an interval of 2 weeks with or without liposomal antigens and 25 µg of MPL-TDM (Sigma) in a total volume of 100 µl/animal/dose (Table S2, supporting information). Groups 1, 2 and 3 received 2.5 µg of rCPA (CPA/CL+MPL), rCPB (CPB/CL+MPL) and rCPC (CPC/CL+MPL) respectively, entrapped in cationic liposome plus MPL-TDM. Group 4 was immunized with the cocktail of liposomal rCPA, rCPB and rCPC (2.5 µg of each protein) plus MPL-TDM (CPA/B/C/CL+MPL). Control groups received PBS or empty liposomes plus MPL-TDM. Ten days after the booster, five animals/group were subjected to analysis of the cellular and humoral responses after immunization (Table 2, supporting information). The remaining hamsters were challenged intracardially with 2.5×10^7^ freshly transformed stationary-phase promastigotes in 200 µl PBS [20]. Immunological assays were carried out post immunization and after 2 and 3 months of challenge infection.

### Measurement of body and organ weight of immunized animals

The total body weight and that of liver and spleen (upon sacrifice) were measured for all immunized groups on days 0, 60 and 90 days post challenge.

### Delayed type hypersensitivity response (DTH)

Delayed type hypersensitivity (DTH) was determined 1 week after boost and at 2 and 3 months post infection. DTH was evaluated by measuring the difference in the footpad swelling at 24 h following intradermal inoculation of the test footpad with 5 µg of either rCPA, rCPB, or rCPC in a total volume of 50 µl, and the swelling of the control (PBS injected) footpad with a constant pressure caliper (Starrett Company, Athol, MA). For cocktail immunized and control groups, DTH response was evaluated by injecting the test footpad with a mixture of rCPA, rCPB, and rCPC (total 5 µg).

### Determination of rCPA, rCPB and rCPC -specific antibody response

The levels of antigen-specific serum IgG and its isotypes, IgG1 and IgG2, were determined in serum samples from experimental hamsters 10 days after the booster and at 2 and 3 months after infection, by ELISA. In brief, 96-well microtiter plates (Maxisorp, Nunc, Naperville, IL) were coated overnight at 4°C with rCPA/rCPB/rCPC (5 µg/ml) singly or in combination (total 5 µg/ml) for controls and cocktail vaccinates, diluted in 0.02 M phosphate buffer (pH 7.5). For total specific IgG determination or IgG subtyping, HRP conjugated goat anti-hamster IgG (1∶8000, Southern Biotech) or biotin-conjugated monoclonal mouse anti-hamster IgG1 and IgG2 (1∶1000, BD Pharmingen, San Diego, CA) were used as secondary antibodies. The plates were blocked with 1% BSA in PBS at room temperature for 3 h to prevent nonspecific binding. The antigen-antibody reaction was detected as described [26].The absorbance was measured using an ELISA plate reader (Thermo, Waltham, MA) at 450 nm.

### Evaluation of in vitro macrophage infection

Two weeks post immunization, macrophages (MΦ) collected from peritoneal exudates of vaccinated hamsters were allowed to adhere to glass cover slips in 0.5 ml RPMI 1640 media containing 10% FCS at 37°C in 5% CO_2_ as detailed elsewhere [26]. MΦ were infected with promastigotes on glass cover slips (22 mm^2^; 10^6^ MΦ/coverslip) at a ratio of ∼10 parasites/MΦ. The unphagocytosed parasites were removed by washing with warm PBS, and the infected MΦ were further incubated in complete medium for 4, 16, 24, 48 and 72 h at 37°C in 5% CO_2_. The cells were then fixed in methanol followed by staining with Giemsa for determination of intracellular parasite numbers. Prior to fixation, culture supernatants were removed at the above mentioned time points and frozen at −70°C for NO analysis.

### Measurement of nitric oxide (NO) and reactive oxygen species (ROS)

The production of nitric oxide (NO) in MΦ culture supernatants was determined using the Griess reagent and the results were expressed in µM nitrite [26]. Briefly, 100 µl of macrophage culture supernatants were mixed with an equal volume of Griess reagent (1% sulfanilamide and 0.1% N-1-naphthylethy-lene diamine hydrochloride in 50% H_3_PO_4_) and incubated at room temperature for 10 min. Absorbance was then measured at 540 nm.

Intracellular reactive oxygen species (ROS) generation was measured using the oxidant sensitive green fluorescent dye 2′,7′-dihydrodichlorofluorescein diacetate (H2DCFDA) (Molecular Probes) [30]. For the experiments, differently treated and untreated peritoneal macrophages were pre-stained with 10 µM H2DCFDA (stock 10 mM solution in DMSO) for 30 minutes in serum-free medium, followed by washing twice with fresh RPMI. Fluorescence measurements were made using a microplate reader (Synergy H1, BioTek; Excitation: 485 nm; Emission: 528 nm), in triplicate and calculated as percent of control.

### Cell proliferation assay

The spleen cells were aseptically removed from the immunized hamsters 2 weeks after the last immunization and single cell suspensions were prepared in RPMI 1640 as detailed elsewhere [11]. The splenocytes were then washed twice, resuspended in the culture medium and viable mononuclear cell number was determined by Trypan blue exclusion [31]. The splenocytes were labeled with carboxyfluorescein succinimidyl ester (CFSE) (Molecular Probes) using a slightly modified technique, originally devised by Lyons *et al.* [32]. Briefly, 1×10^7^ cells/ml were incubated with 2 µM CFSE for 10 min at 37°C. The labeling was quenched by adding one volume of cold PBS and washed twice in cold RPMI 1640 (Sigma). Then the cells were cultured in triplicate in 24-well flat bottomed tissue culture plates (Nunc, Roskilde, Denmark) at a density of 1×10^6^cells/well in a final volume of 1 ml and stimulated with either rCPA, rCPB or rCPC (5 µg/ml) singly for single antigen immunized groups or in combination (5 µg/ml total) for controls and cocktail vaccinates or conA (2.5 µg/ml). After five days, the cells were collected and analysed on a FACSCanto flow cytometer (Becton Dickinson) using the FACSDiva software.

### Cytokine response

Complementary DNA was synthesized using SuperScript III First-Strand Synthesis kit (Invitrogen, Grand island, USA) from 100 ng of total RNA isolated from splenocytes of differently vaccinated hamsters using the RNeasy mini kit (Qiagen), before and after 2 and 3 months of infectious challenge. Measurement of levels of IL-2, TNF-α, IFN-γ, IL-4, IL-10 and TGF-β was carried out from splenocytes of vaccinated and control animals by a two-step SYBR green I real-time reverse transcription PCR using specific primers (Table S3, supporting information) as reported earlier [33]–[35]. Conventional PCR using 100 ng of hamster DNA, 300 nM cytokine specific primers and High Fidelity PCR Enzyme Mix (Fermentas) was previously carried out in order to optimize the real time PCR for each target. Hamster HGPRT (Hypoxanthine-guanine phosphoribosyltransferase) was used as an endogenous control to avoid variations between the samples. The cDNA samples were subjected to an initial incubation for 10 minutes at 95°C and then 40 cycles of 95°C for 15 sec and 58°C for 30 s, and 72°C for 30 s in a 7900 HT Fast Real-Time PCR System (Applied Biosystems). Each measurement was carried out in triplicate. The relative expression of mRNA in terms of fold change was calculated by the comparative Ct (2^−ΔΔCt^) method [36] normalized to HGPRT expression. A no-template control without genetic material was included to eliminate nonspecific reactions.

### Histopathology of livers of experimental animals

A part of liver from the sacrificed hamsters (2 months post challenge) was used for tissue histology. After standard procedures like paraffin embedding, histological sectioning and mounting on glass slides, sections were stained with hematoxylin and eosin. Sections from each liver (n = 3 hamsters per group) were examined by counting 25 consecutive 40× microscopic fields per section.

### Evaluation of parasite burden in liver and spleen

After 2 and 3 months of challenge infection, the animals were sacrificed to determine the parasite load in liver and spleen. The course of organ parasite load was monitored by the microscopic examination of Giemsa-stained impression smears of liver and spleen, expressed as Leishman Donovan Units (LDU) [37] as well as by limiting dilution assay (LDA) [38] as described previously. For LDA, a weighted piece of liver and spleen isolated from different vaccinated groups were homogenized and five-fold serial dilutions of homogenized tissue suspension were cultured at 22°C for 21 days in 96-well tissue culture plates (Nunc). The culture plates were examined every 7 days for the presence of motile promastigotes for 21days. The reciprocal of the highest dilution that was positive for viable parasites was considered to be the concentration of parasites per mg of tissue. The total organ parasite burden was calculated using the weight of the respective organs.

### Statistical analysis

One-way ANOVA statistical test was performed to assess the differences among various groups. Multiple comparisons Tukey-Kramer test was used to compare the means of different treatment groups using the GraphPad Prism 5.0 software for windows (http://www.graphpad.com). The Kaplan-Meier method was used to estimate survival rates, and the Log-rank test was applied to compare the survivalities between different groups of hamsters. A value of *p*<0.05 was considered to be significant for all analyses.

### Accession numbers

Cysteine protease A (KF018070)

Cysteine protease B (KC609324)

Cysteine protease C (JX968801.1)

## Results

### Cloning of *L. donovani cpa*, *cpb* and *cpc*

**Figure 1:**
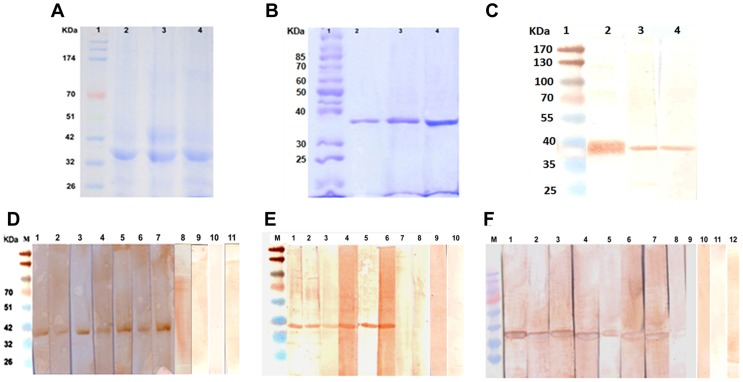
Cloning and expression of *cpa, cpb and cpc*. A, Coomassie blue stained protein fractions showing overexpression of *cpa*, *cpb* and *cpc* in *E. coli* BL21 (DE3). Lane 1, prestained molecular weight marker; lane 2, *cpa*; lane 3, *cpb*; lane 4, *cpc*. B, Coomassie blue staining of 10% SDS-PAGE showing purified proteins. Lane 1, molecular weight marker; lane 2, rCPA; lane 3, rCPB and lane 4, rCPC. C, recombinant proteins probed with anti-polyhistidine antibody after Western blot. Lane1, molecular weight marker; lane 2, rCPA; lane 3, rCPB and lane 4, rCPC. D–F, Plasma recognition of *L. donovani* CP. Immunoblot analysis of recognition of recombinant CPA (D), CPB (E) and CPC (F) by human blood plasma from infected (odd numbered lanes 1–7) and cured (even numbered lanes 1–7) individuals suffering from VL. Lane 8–12, endemic controls. M, molecular weight marker.

Full length *cpa, cpb and cpc* were successfully cloned in the right orientation in the bacterial expression vector pET28a and the overexpressed proteins from *E. coli* BL21 (DE3) cells were purified under denaturing conditions (Fig. 1 & S2). The molecular weights of rCPA, rCPB and rCPC were approximately 38.8, 38.5 and 37.4 kilodalton (kDa) respectively. The yield of purified proteins was approximately 3–5 mg per liter of culture. ClustalW analysis shows 98–99% sequence homology of these three CP with the different strains of *L. donovani* and *L. infantum*. Also, *L. donovani cpa*, *cpb* and *cpc* were highly similar also to those of *L. major cpa*: AJ130942.1 (64% identity), *cpb*: U43706.1 (88% identity) and *cpc*: XM_003722109.1 (95%) respectively. Phylograms depicting the phylogenetic relationships of *cpa*, *cpb* and *cpc* among different *Leishmania* species are shown in Fig. S1 (supporting information). After successful entrapment in cationic liposomes, the level of incorporation ranged between 70–80%. Protein integrity after liposomal encapsulation was evaluated by 10% SDS-PAGE followed by Coomassie staining. Positive reactions of all three recombinant proteins CPA, CPB and CPC with anti-His monoclonal antibody in Western blot analysis identified these proteins from bacterial lysates (Fig. 1C).

### Recombinant CPs are highly immunoreactive in cured and active VL patients

To determine the presence of antibodies against *L. donovani* CPs in human VL, rCPA, rCPB and rCPC were subsequently blot checked against a panel of plasma samples from kala-azar patients. Blood plasma isolated from patients infected with *L. donovani* recognized all the three recombinant proteins (rCPA, rCPB and rCPC) separately at 1∶1000 dilution (Fig. 1, E–G). This finding verified the reactivity of the selected proteins in both active and cured VL patients, but not in healthy controls. Recognition of *L. donovani* CPs also by Brazilian VL (*L. chagasi*) patient plasma (data not shown) is predictive of cross-protective potential of these vaccine candidates and also for diagnosis of kala-azar.

### Characterization of liposomal delivery system

**Figure 2:**
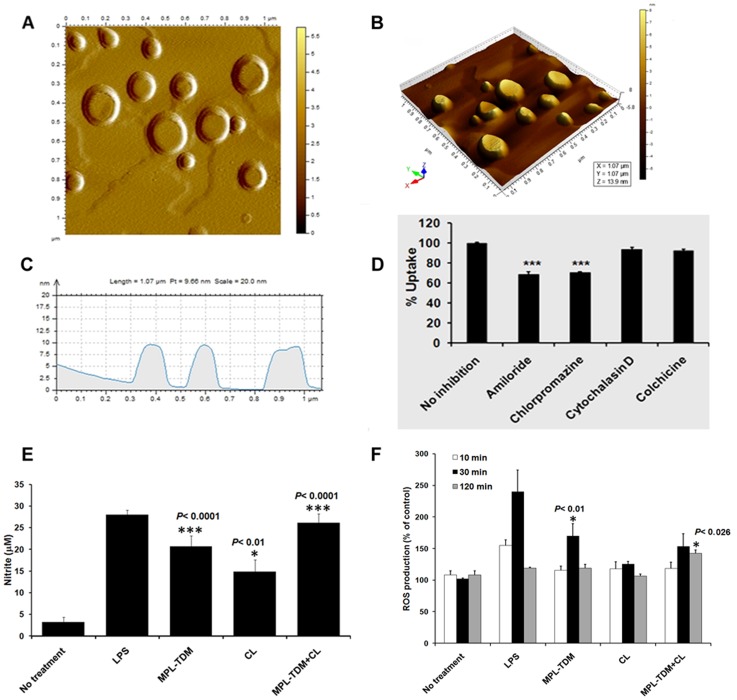
Particle characterization, cellular uptake and localization of cationic liposomes in hamster macrophages (MΦs). A–C, tapping mode AFM images of liposomes obtained 15 mins after deposition on mica support. A, AFM image presented as two-dimensional graphics showing the clean spherical shaped DSPC-liposomes. Amplitude-flattened views of liposomes are shown. B, 3D images of DSPC-liposomes by AFM study. C, horizontal cross sections indicating the height of the liposomes from the substratum, i.e., the mica sheet. D, Elucidation of cellular uptake mechanisms of cationic DSPC-liposomes in MΦs. Percent uptake of Rh-123 (green) labeled liposomes is shown in hamster peritoneal MΦs treated with or without different biochemical inhibitors, studied by flow cytometry. Data represent the mean and standard deviation of three different experiments performed in duplicate (***p<0.001, compared to no-inhibitor control). E–F, Activation of innate response towards cationic liposomes and MPL-TDM. Hamster peritoneal MΦs were isolated and stimulated with LPS (1 mg/ml), liposomes (50 mM), MPL-TDM (100 ng/ml) and combination of liposomes (50 mM) and MPL-TDM (100 ng/ml). The release of NO (E) and ROS (F) were measured subsequently. The data are representative of three independent experiments with similar results.

AFM in the acoustic alternative current mode allows the observation of the liposomal surface morphology and structure, overcoming sample manipulation (Fig. 2). Flattening of vesicles on the mica support few minutes after deposition indicates a moderate stability of the liposomes. AFM images clearly depict the spherical, well-defined shape of the liposomes with visible multilamellar structures and heterogeneous size distribution, which is also maintained after protein loading (rCPC taken as reference) (Fig. S4). Size of the vesicles ranged between 137–172 (±14.3) nm.

### Cellular uptake and antigen delivery by cationic liposomes

Among all APCs, MΦs play a central role in processing of liposomal antigens in lysosomes for successful Ag presentation to T cells [39]–[44]. To expand on that, we investigated intracellular trafficking patterns of our cationic liposomes as well as their mechanism of internalization in hamster peritoneal MΦ in vitro (Fig. 2D & S3). LysoTracker Red was used as a fluorescent acidotrophic probe for tracking acidic organelles in MΦ. Confocal laser scanning microscopy (CLSM) of MΦ cells incubated for 2 hrs with both Rh123-labeled liposomes (green), LysoTracker Red (red) and DAPI(blue) simultaneously showed successful co-localization (yellow) of labeled liposomes in acidic compartments such as lysosomes (Fig. S3, B) which confirms antigen presentation by MΦ. For a quantitative insight into the various potential mechanisms of endocytosis, a series of liposome uptake assays were performed in the presence of different inhibitors (amiloride, chlorpromazine, cytochalasin D and colchicines) to block each individual pathway. The use of amiloride (macropinocytosis inhibitor) and chlorpromazine (clathrin-mediated endocytosis inhibitor) strongly inhibited the uptake of cationic liposomes by ∼31% (±2.674) and ∼29% (±1.07) respectively (Fig. 2D). We deduce partial overlap or synergistic involvement of these pathways for liposomal uptake in hamster peritoneal MΦ. By contrast, cytochalasin D and colchicines had only minimum effect on the cellular entry of liposomes indicating minor contributions of actins and microtubules in the uptake process. Nevertheless, other unidentified pathway(s) of endocytosis may also be involved in liposome trafficking through MΦ. The FACS estimation of the inhibitory effects on internalization and post-endocytic trafficking of labeled liposomes further confirmed the CLSM data (Fig. S3, A).

### Cationic liposomes with MPL-TDM activates the MΦ innate immunity by NO and ROS-dependent mechanisms in vitro

To evaluate innate capacity of our adjuvant formulation to stimulate MΦs, naïve peritoneal MΦs were isolated and stimulated with bacterial lipopolysaccharides (LPS) (1 mg/ml), cationic liposomes (50 mM), MPL-TDM (100 ng/ml) and combination of cationic liposomes (50 mM) and MPL-TDM (100 ng/ml) (Fig. 2, E & F). MPL-TDM alone activates MΦs from naïve hamsters resulting in increased NO and ROS production, which are regarded as the potent microbicidal molecules responsible for parasite clearance from infected MΦs [45]. Evidence for such endotoxin and lipid A mediated NO production via inducible iNOS is increasing [46], [47] which results in innate host cell activation and subsequent adjuvant properties. The significant production of NO (Fig. 2E) and ROS (Fig. 2F) in response to cationic liposomes along with MPL-TDM are in agreement with our previous study which showed that stimulation of murine DCs with liposomes plus MPL-TDM could enhance IL-12 (p40) and NO generation in vitro [11]. These data indicated that combining MPL-TDM with cationic liposomes result in increased the adjuvant activity of the formulation on the APCs in vitro, serving our rationale for its utilization.

### DTH and antibody response as a measure of vaccine induced protection

**Figure 3:**
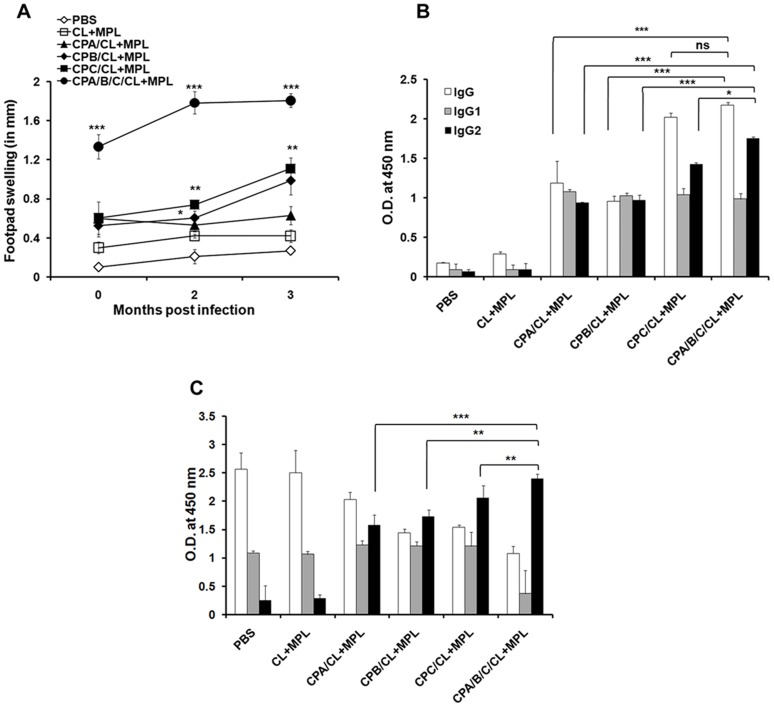
Pre and post challenge cellular and humoral responses in vaccinated hamsters. A, DTH response before and after 2 and 3 months post infection. B, serum IgG, IgG1 and IgG2 at 10 days after final vaccination. C, IgG, IgG1 and IgG2 response after 3 months post infection. Data are presented as the mean absorbance at 450 nm ± S.E., ***, *p*<0.0001, ns = not significant. Asterisks over each bar indicate significant differences in comparison to adjuvant controls. Asterisks over line indicate significant differences between groups. *, *p*<0.05; **, *p*<0.01. The results are mean ± S.E. of five individual hamsters per group, representative of two independent experiments with similar results.

DTH was measured as an index of in vivo elicitation of cell-mediated immunity. All the CP immunized hamsters displayed steady increase in DTH response, both before and 2 and 3 months after infection. Among the three antigens, liposomal rCPC vaccination induced maximum DTH followed by rCPB and rCPA at 3 months post challenge. Highest DTH was obtained for cocktail vaccinates (Fig. 3A; *p*<0.0001compared to free adjuvants), which was even significantly higher than rCPC (*p*<0.0001) vaccinated hamsters post vaccination as well as at 2 and 3 months post infection. Serum samples were analyzed for total IgG, IgG1 and IgG2 both before and after infection in vaccinated animals. Although distinct IFN-γ and IL-4 mediated IgG isotype switching is not reported in hamsters, IgG1 and IgG2 are still considered as surrogates of Th2 and Th1 response similar to murine IgG1 and IgG2a/b respectively [48]. Shown in Fig. 3 are the antibody levels in immunized (Fig. 3B) and 3 months post challenged (Fig. 3C) hamsters (sera dilution 1∶1000). In comparison to controls, significant enhancement of antigen-specific total IgG, IgG1 and IgG2 with a dominance of IgG2 was observed in all antigen immunized hamsters. Contrastingly, after infection, the levels of IgG2 were quite low in hamsters receiving PBS or free adjuvants as controls indicating a Th2 response favouring disease progression. All CP immunization induced higher specific IgG2 titers than IgG1, with significant difference observed between the IgG2 subclasses in single antigens and cocktail vaccination schedule (Fig. 3B). Remarkable IgG2 dominance was observed in hamsters receiving rCPC and triple antigen cocktail which also differed significantly between rCPC and cocktail immunized groups (*p*<0.01) (Fig. 3B & C). The results were consistent with lymphoproliferation and Th1 biased cytokine response, which showed strong CMI response in animals receiving rCPC and cocktail antigens.

### Mitogenic and antigen specific lymphoproliferation in vaccinated hamsters

**Figure 4:**
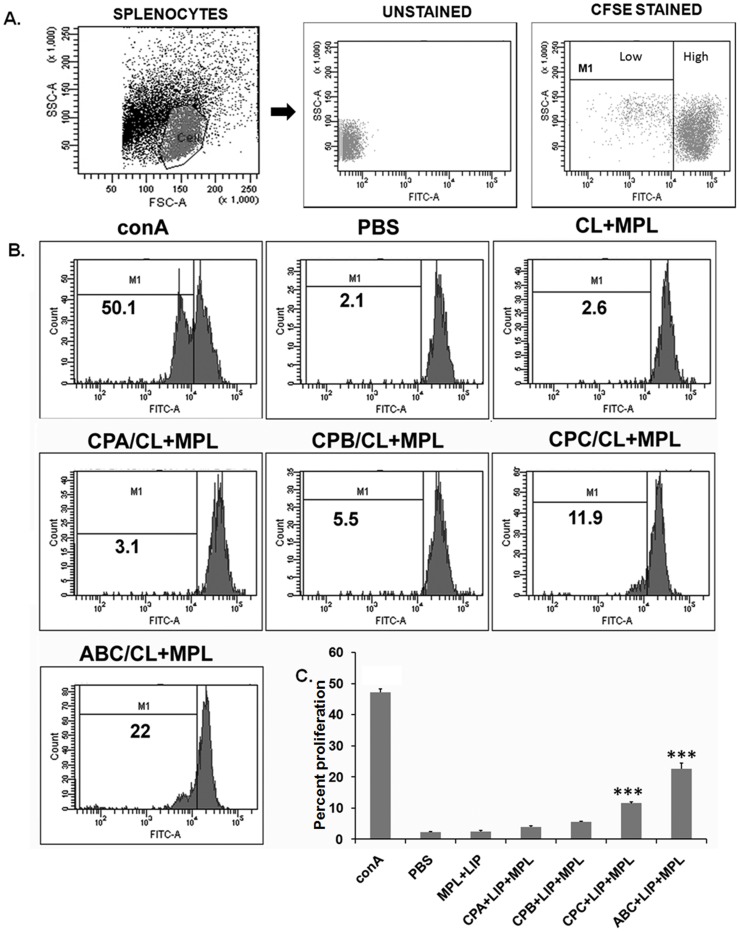
Proliferative response in hamsters vaccinated with liposomal CPs with MPL-TDM. Splenocytes from immunized animals were labeled with CFSE (2 µM) and restimulated in vitro for 5 days with 5 µg/ml of specific antigen or 2.5 µg/ml ConA. Antigen-specific splenocyte proliferation of individual animals was analyzed by flow cytometry and CFSE dilution on gated cells. A, Gating strategy for hamster lymphocytes and CFSE ^high^ and CFSE ^low^ populations. B, Representative histogram showing percent proliferation, calculated in the indicated region (bar). C, the bar graphs show mean percent proliferation of the lymophocytes, which is inversely proportional to cell divisions. The data are representative of one animal from two experiments with at least three hamsters in each group. Significant differences between means are indicated: *, *p*<0.05; **, *p*<0.01 compared to PBS controls.

Impaired T-cell mediated immunity as assessed by in vitro lymphocyte proliferation has been the hallmark of progressive VL [49], [50]. Proliferative capacity is a wanted feature of vaccine, reflecting stimulation of T cell responses. Therefore, to gain insight into cellular immunity developed in hamsters after vaccination, antigen specific splenocyte proliferation was evaluated using CFSE. As shown in Fig. 4, ConA (mitogen), taken as a positive control, highly enhanced cell proliferation. Splenocytes from all the CP vaccinated hamsters proliferated in response to corresponding antigen. Among the three CPs, rCPC showed higher antigen-specific proliferation compared to rCPA and rCPB. Significant enhancement of antigen-specific splenocyte proliferation was observed for liposomal rCPC (*p*<0.001) and liposomal cocktail (*p*<0.0001) vaccinated animals compared to adjuvant controls. Highest percent lymphoproliferation was observed in hamsters vaccinated with triple antigen cocktail which was significantly higher than rCPA (*p*<0.0001), rCPB (*p*<0.0001) and rCPC (*p*<0.0001) as single antigen. Taken together, these finding suggest that all three CPs present in the cocktail vaccine act synergistically to counteract the impaired T cell response after challenge for improved protection.

### In vitro antileishmanial activity of peritoneal macrophages

**Figure 5:**
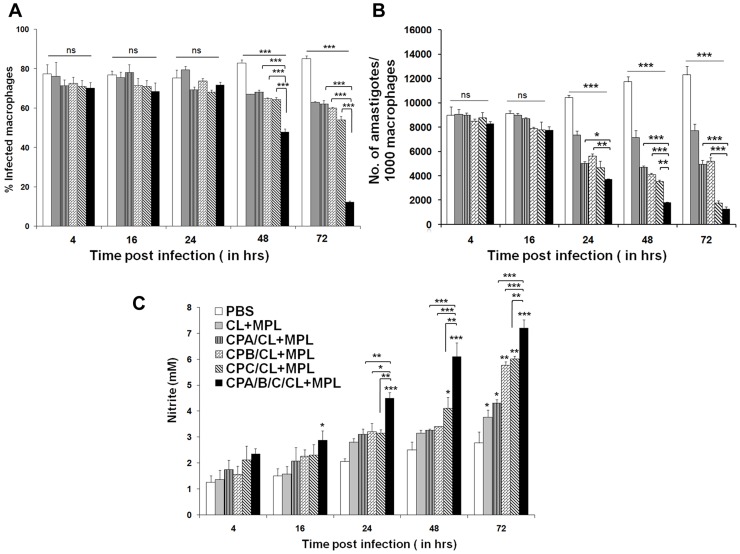
Anti-leishmanial activity of macrophages from liposomal CP immunized hamsters. A, Percentages of infected peritoneal MΦs from different groups of hamsters from different liposomal CP immunized groups after infection with *L. donovani* promastigotes. B, Total number of amastigotes/1000 peritoneal MΦs in vitro after immunization with liposomal cysteine proteases after 4 h, 16 h, 24 h, 48 h and 72 h infection with *L. donovani*. C, NO production by infected MΦs after 4 h, 16 h, 24 h, 48 h and 72 h of in vitro infection. The results are mean ± S.E. of five individual hamster per group, representative of two independent experiments with similar results. Statistically significant (*, *p*<0.01; **, *p*<0.001;***, *p*<0.0001) differences in MΦ infection, parasite multiplication and nitrite production were compared to PBS controls. Asterisks over line indicate significant differences between groups.

MΦ have long been known to play an important role in leishmanicidal activity through IFN-γ and IL-12 mediated production of NO [51]. However, like human, NO generation in infected hamsters is severely impaired due to lack of IFN-γ mediated upregulation of nitric oxide synthase (NOS) 2 mRNA [52]. To evaluate the vaccine-induced activation of MΦ to arrest parasite multiplication, the number of intracellular amastigotes and NO production were determined in resident peritoneal MΦ from different immunized groups at designated time points (4 hr, 16 hr, 24 hr, 48 hr and 72 hr) after in vitro infection with *L. donovani* (Fig. 5). No significant difference was observed between groups in the numbers of initial uptake and percent infected MΦ till 24 h of incubation (Fig. 5A). However, after 48 and 72 h, significant leishmanicidal activity, lowering of percent infected MΦ (Fig. 5A) and mean number of amastigotes/MΦ (Fig. 5B) was observed in all CPs immunized animals compared to controls. Of interest, maximum inhibition of parasite multiplication in vitro was observed in hamsters receiving rCPC and cocktail antigens (*p*<0.0001) which was even significantly higher than liposomal rCPA (*p*<0.0001) and rCPB (*p*<0.0001) vaccinates, sustained upto 72 h of incubation (Fig. 5B). However, no significant difference in amastigote killing activity was noted between rCPC and cocktail vaccinates after 72 h. This may be due to the reason that control of in vitro infection by MΦ is not solely due NO production but also by the milieu of cytokines and chemokines, specially IL-12 and TNF-α secreted by the parasitized APCs [53]. Almost comparable levels of these may also account for the lack of significant difference in parasite clearance between rCPC and cocktail vaccinates in vitro. NO level in single antigen vaccinates were almost comparable without significant difference between the groups upto 48 h, but was significantly upregulated in rCPB and rCPC vaccinated animals at 72 h post infection compared to controls (Fig. 5C). Interestingly, MΦ from liposomal cocktail antigen immunized animals released the highest amount (7 µM) of NO which was ∼4.3 fold more than that of infected controls and ∼2-fold more than the single antigen (rCPA, rCPB, rCPC) immunized groups after 72 h post culture. Collectively, immunization with liposomal cocktail CPs mounted highest antileishmanial activities towards *L. donovani* infected MΦ, followed by liposomal rCPC>rCPB>rCPA, immunized animals.

### Histopathological studies

The degree of cellular response in terms of granuloma formation in liver of immunized and infected control hamsters indicates the histological features of either parasite clearance or multiplication in this organ [54]. Considerable variation among animals between different groups was observed in hepatic histology 2 months after *L. donovani* challenge (Fig. S6). In the infected controls the cellular response in liver was restricted to few immature granulomas, comprising of fused infected Kupffer cells with a large number of infiltrating lymphocytes and histiocytes. The histological features in controls were indicative of suboptimal cellular response inappropriate for parasite clearance. Occasional portal tract inflammation, fibrosis and hepatocyte degeneration resulting in loss of normal tissue architecture were also visible in liver of infected controls. Mature granulomas with compact collection of cellular infiltrates, indicative of optimal cellular response leading to parasite clearance, were mostly seen in groups immunized with cocktail and liposomal rCPC vaccinated animals (Fig. S6, A). Figure S6, B shows a magnified view of a representative granuloma assembly in a cocktail immunized hamster, showing cellular infiltration of lymphocytes and monocytes.

### Vaccination with CP cocktail generates optimum protection in hamsters against *L. donovani* challenge

**Figure 6:**
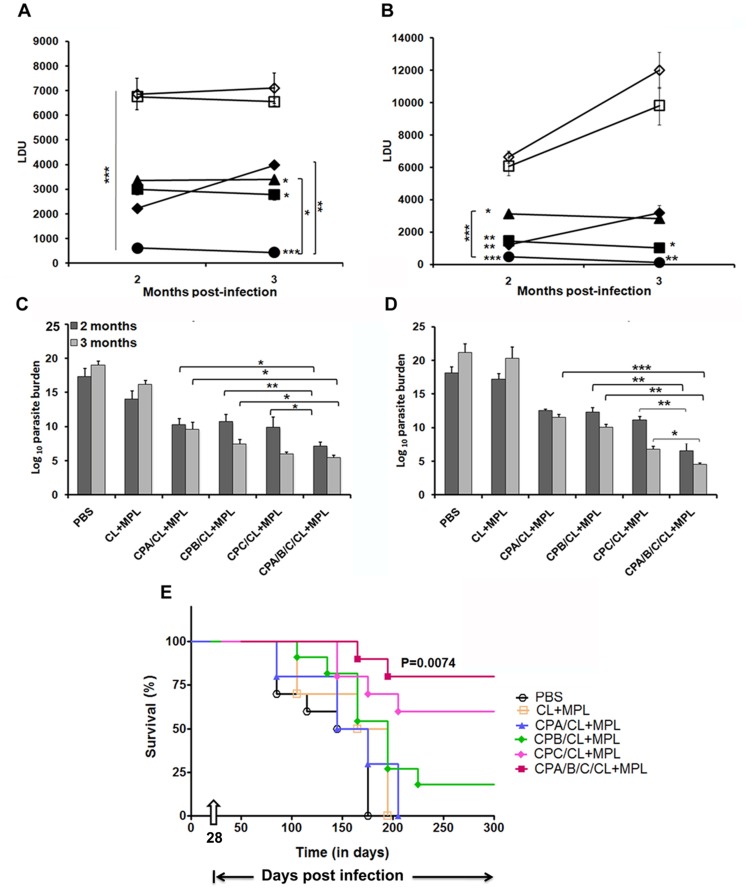
Protection and survival against *L. donovani* infection in immunized hamsters. A–D, kinetics of liver (A) and spleen (B) parasite burden by LDU, liver LDA (C) and spleen LDA (D) of hamsters 2 and 3 months after intracardiac challenge of 2×10^7^ virulent *L. donovani* promastigotes. Results are expressed as log of total organ parasite burden. Data represent the mean ± S.E. (n = 5/group). ***p<0.001 compared to adjuvant controls. Asterisks over line indicate significant differences between groups. E, Kaplan-Meier survival curves comparing survivality in different groups of vaccinated hamsters. Survival results are from 10 hamsters per group. Survival distribution was analyzed by log-rank (Mantel-Cox) test, and level of significance is indicated by P value.

Weight loss and hepatosplenomegaly in infected controls, as a normal course of progressive VL was evident from 2 months of infection (Fig. S5). Fig. 6A–D shows the outcome of vaccination on parasite burden in liver and spleen at 2 and 3 months post infection. In all the CP vaccinated groups, parasite load in spleen sharply decreased after 2 and 3 months of infection compared to controls. At 3 months post infection, significantly high yet comparable level of protection was induced by rCPA and rCPB immunized groups, suggesting a specific partial protection induced by these antigens compared to controls (*p*<0.0001). rCPC induced greater protection than both rCPA and rCPB, when injected alone. Of interest, the mean hepatic and splenic parasite burden (LDU) were lowered by ∼93% and ∼98% respectively, in animals immunized with cocktail antigens as compared to the adjuvant controls at 3 months post infection. Significantly higher protection was obtained using the cocktail vaccine compared to the single antigens: rCPA (73%), rCPB (76%) but not rCPC (91%) in spleen. Our result obtained in LDU was reconfirmed through limiting dilution assay, LDA (Figure 6, C & D), the more sensitive method for monitoring viable parasites. Free adjuvant (CL+MPL) itself generated some protection resulting in 2.46 log10±.61 in the liver and 1 log10±1.66 in the spleen at 3 months post challenge. This was, however, not significant compared to PBS controls. After 3 months post infection, hamster immunized with liposomal rCPA and/rCPB showed reduced parasite burden ∼6.5–9 log-folds in liver and ∼8.8–10 log-folds in spleen, respectively, compared to animals immunized with adjuvant alone (Figure 6, C & D). Immunization with rCPB thus resulted in better protection than rCPA in clearing the parasites from visceral organs. Interestingly, hamsters vaccinated with liposomal rCPC showed a parasitic burden of 5.963 log10±0.269 in the liver and 6.77 log10±0.463 in the spleen, with an impressive 4 (log10)-fold decrease in the parasite burden compared to hamsters receiving liposomal rCPA (9.55 log10±1.037 in the liver and 11.5 log10±0.467 in the spleen, *p*<0.0001) and 2 (log10)-fold reduction compared to liposomal rCPB (7.413 log10±0.016 in the liver and 10.02 log10±0.451 in the spleen, *p*<0.0001). Highest level of protection against *L. donovani* was however achieved in hamsters immunized with the liposomal triple antigen cocktail which showed 13 log-fold and 16 log-fold reductions in the parasite burden in liver and spleen, respectively, compared to adjuvant controls (*p*<0.0001) after 3 months of infectious challenge. Moreover, they exhibited minimal weight loss and the surviving animals appeared apparently healthy till the termination of the experiment. Consequently, almost 80% survival was observed in hamsters injected with the cocktail vaccine for at least 180 days post infection (Fig. 6E). In contrast, almost 50–60% of the controls died after 3 months due to steady increase in parasite load (Fig. 6E).

### In-vivo immunomodulation

**Figure 7:**
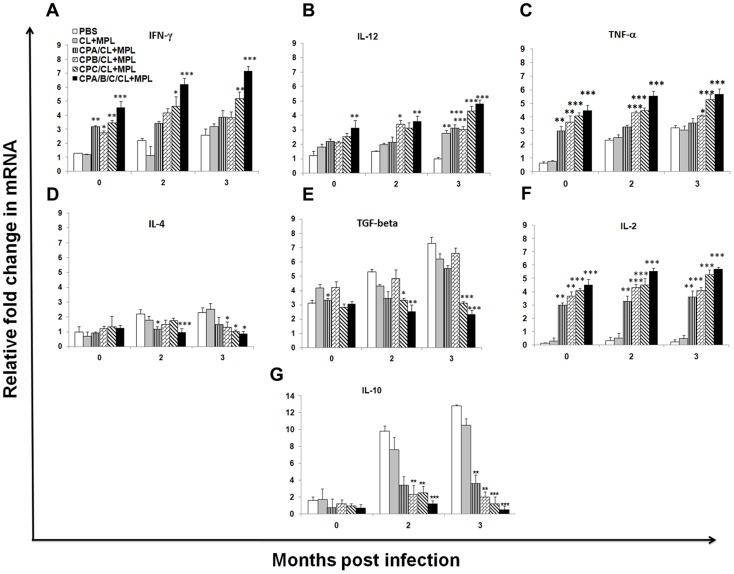
Th1/Th2 cytokine profiles of immunized hamsters at different time intervals pre- and post-infection by quantitative real-time–PCR. Fold change in mRNA expression profiles of A, IFN-γ; B, IL-12; C, TNF-α; D, IL-4; E, TGF-β; F, IL-2; G, IL-10. Each gene was normalized to the housekeeping gene (Hypoxanthine-guanine phosphoribosyltransferase, HGPRT) to avoid variations between different samples. Results are expressed as the mean ± S.E. of five hamsters individually assayed from each group. *, *p*<0.05; **, *p*<0.01;***, *p*<0.001 assessed by one-way ANOVA and Tukey's multiple comparison tests.

In experimental VL, the efficacy of antileishmanial prophylaxis chiefly depends on CMI and balanced production of endogenous Th1 and Th2 cytokines [55]. The cytokine expression profiles of vaccinated and control infected hamsters were thus dissected to find out their role in immunoprotection. All immunized groups receiving either of liposomal rCPA, rCPB and rCPC singly or as cocktail produced significant upregulation of IL-2 and IFN-γ mRNAs in comparison to controls, which progressively increased at 2 and 3 months post infection (Fig. 7A). rCPA and rCPB immunizations although showed heightened IFN-γ and IL-12 expression, also enhanced expression of disease promoting cytokines like IL-4 and IL-10 mRNAs after infection, which perhaps limited their vaccine efficacy. The splenocytes of vaccinated hamsters receiving liposomal rCPC and cocktail of three liposomal CPs showed higher IFN-γ, IL-2, TNF-α and IL-12 mRNA expression than all other groups but appreciable downregulation of macrophage deactivating cytokines like IL-4, IL-10 mRNAs, before and after infection (Fig. 7). Even after 3 months post infection, an appreciable ∼4.5 fold and ∼3.5 fold upregulation of IFN-γ (*p*<0.001) and IL-12 (*p*<0.001) mRNAs respectively were observed in cocktail immunized animals compared to PBS controls. There were 9 to10-fold reductions in IL-10, for rCPC and cocktail immunized groups (*p*<0.0001) after 3 months post infection (Fig. 7 D) compared to adjuvant controls which was directly related to disease progression. Further analysis of the induced cytokine expression by the means of IFN-γ/IL-10 mRNA ratio (not shown) revealed that liposome formulated cocktail CP vaccines clearly induced a strongest Th1 response in comparison to all other groups, required for sustained protection. Further, augmented level of disease-resolving cytokine, TNF-α mRNA (Fig. 7, C & F), notably high in rCPB, rCPC and cocktail vaccinated groups corroborate intracellular parasite killing. In particular, a dramatic shift towards Th1 phenotype and most probably an effective CMI response was maintained by high IL-12 and IL-2 mRNA levels even at 3 months post infection in cocktail vaccinates. Although we could not dissect the T cell phenotype responsible for protective immunity, results from antigen-specific T-cell proliferation, NO production, increased mRNA expression of IL-2, IL-12, IFN-γ, robust DTH responses, and IgG2 antibody titer are indicative of durable T-cell response which is sustained at least up to 3 months post infection. Further, our results emphasize a major role of IL-10 and IL-4 upregulation for disease progression and death in infected controls, probably mediating their effect through IFN-γ blocking, macrophage deactivation and lymphocyte apoptosis as reported earlier [56], [57].

## Discussion

Till date, ‘leishmanization’ remains the gold standard for vaccination against leishmaniasis for providing long term protection. However, safety concerns and anti-vector immunity have complicated the development of live vaccines against *Leishmania* [58]. In contrast, a non-living subunit vaccine containing selected antigens combines safety and rational designing to target the intracellular pathogens, avoiding anti-vector immunity. However, subunit protein vaccines without adjuvant often suffer from limited or non-existent T cell response due to poor antigen presentation via APCs [59], [60]. Interestingly, current prophylactic vaccine strategies against intracellular pathogens like *Leishmania* focus on strengthening host innate immunity that target the pathogen, in addition to vaccine induced adaptive response [61]. Vaccination against *Leishmania* requires both innate and adaptive arms of host defence mediated through MΦ, dendritic cells and both CD4^+^ and CD8^+^ T cells for protection [62], [63]. The liposomal cargos loaded with protein antigens in combination with defined immunostimulatory molecules mimicking pathogens in reductionist mode are attractive formulations to elicit protective T cell immunity. Use of TLR agonists with cationic liposomes allows concurrent antigen presentation along with targeting pattern recognition receptor (PRR) pathways, for effective expansion of effector T cells [64], [65]. In this study we therefore selected highly immunostimulating adjuvant formulation (cationic liposomes and MPL-TDM) to investigate the ability of leishmanial CPs to generate both humoral and cell mediated immune response via subcutaneous route.

Lysosomal cysteine proteases (CP) are reported to be involved in distinct cellular functions and make safe vaccine antigens against *Leishmania* [15]–[17]. Nevertheless, evaluation of protective efficacies of these proteins against *L. donovani* is lacking. The present study compares the efficacies of three recombinant cysteine proteases (rCPA, rCPB, rCPC) and their cocktail using cationic liposomes with MPL-TDM adjuvant platform for the first time against *L. donovani*. Hamsters are well-established animal models for *L. donovani* infections, with high innate susceptibility and clinicopathological resemblance to human VL [66], thus suitable for evaluation of vaccines for human use.

Macrophages, the major intracellular niche for *Leishmania*, are also involved in antigen presentation and vaccine-induced leishmanicidal activity, usually predictive of disease outcome in vivo. Phagocytosis by macrophages plays an important role in lymph node retention of liposomes after subcutaneous immunization [67], [68]. Cellular internalization and successful endosomal/lysosomal loading for MHC class II antigen presentation via APCs has been studied for the first time for DSPC bearing cationic liposomes showing involvement of distinct endocytic pathways (macropinocytosis and clathrin-mediated) (Fig. 2) for cellular entry. This is advantageous in lowering the amount of vaccine Ag by liposomal encapsulation enabling administration of higher effective doses if required [26].

Impaired cellular immunity along with a shift towards Th2- type immunity has been the hallmark of VL [50]. Although the role of humoral response in resolution of VL is controversial, elevated level of IgG after infection usually correlates with VL progression in hamsters. The rapid increase in anti-leishmanial IgG observed after antigen immunization appeared to result from associated MPL-TDM which is known to augment humoral in addition to CMI response [69]. However, the sustained dominance of IgG2 over IgG1 in CP vaccinated animals indirectly reflects the Th1-biased protective immune responses at 2 and 3 months post challenge. This is in accordance with enhanced proliferation of hamster splenocytes isolated from cocktail vaccinated group over nonvaccinated and single antigen immunized animals.

In VL, a balance must be established between Th1 and Th2 cytokines for parasite clearance. High IL-10 and IL-4 can lead to disease exacerbation and vaccine failure even in presence of high IFN-γ [70]. TNF-α, another well-defined inflammatory cytokine with antileishmanial properties, is known to act either alone or with IFN- γ to induce the production of NO and ROS and might have a strong additive effect in clearing parasites from vaccinated animals. All three CPs immunizations resulted in increased level of IL-12 driven IFN-γ, but low amounts of IL-4, IL-10 and TGF-β. Thus, enhanced IL-2, IL-12, TNF-α and IFN-γ mRNA expression but downregulation of IL-10, most remarkable in cocktail vaccinated hamsters is chiefly responsible for strong Th1 biased immunity after infection [71]. Interestingly, IL-10 is also generated at low levels after infection as a part of effector response to prevent autoimmunity and maintenance of T cell proliferation [72]. Significantly low but consistent mRNA expression for both IL-4 and IL-10 in all CP vaccinates, more noticeably in rCPC and cocktail immunized groups, probably circumvents uncontrolled Th1 response and tissue damage due to high post-challenge IFN-γ and IL-12 expression levels in these groups. Of interest, hepatic histology, in vitro lymphoproliferation and NO mediated clearance of parasites from MΦ nicely correlates with in vivo protection after virulent challenge. Maximum number of matured hepatic granuloma was observed in rCPC and cocktail immunized hamsters after 2 months which led to almost complete clearance of parasites from visceral organs. The combination of CPA, CPB and CPC resulted in enhanced protection in liver (>93%) and spleen (>98%) which was even higher than all other CPs based vaccines tested against *Leishmania* so far [15]–[17]. Previous vaccine attempts with different liposomal adjuvants have reported remarkable efficacy of about 80–90% protection against *Leishmania* [73] and other pathogens [74], [75]. Liposome entrapped native LD51 (β-tubulin) and LD31 (ATP synthase α-chain) from *L. donovani* induced 75–77% protection in mice, through intraperitoneal route [73]. Similarly, 7–10-log-fold reduction in parasite burden upon was obtained upon vaccination with liposomal recombinant gp63 with MPL-TDM in mice [11]. One plausible explanation for the synergistic enhancement of protection with cocktail CP vaccination is the cumulative increase in T-cell epitopes in a non-antagonistic manner. In this context, it is a known that the parasite CPA, CPB and CPC all belong to the same group of papain-like CPs, and probably behave like a multi-subunit vaccine when given in combination. Enhanced Th1 dominance in the current study chiefly arises from the associated MPL-TDM within the liposomal formulation, increasing the efficacy and adaptability of the delivery system as reported earlier in mice [11]. MPL is known for its direct interaction with TLR4 on dendritic cells (DC) influencing IL-12/IFN-γ axis, to skew the T cell response towards a Th1-phenotype [76], [77]. Taken together, vaccination with liposomal CPs combined with MPL-TDM confers a Th1 biased mixed Th1/Th2 response to reduce the parasite burden in hamsters. Although, direct comparison of our work with previous reports is difficult, it is noteworthy that rCPC individually induced better protection than promising vaccine candidates like gp63, ORFF, LelF-2 tested in murine VL [78] against *L. donovani*. Hence, we propose that combining rCPC or its antigenic motif with other immunodominant antigens as cocktail or fusion hybrid can induce durable and complete protection against VL. Here we report almost complete elimination of parasites from both liver (16-log fold) and spleen (13-log fold) after vaccination with CP triple antigen cocktail and *L. donovani* virulent challenge. Though sterile protection was not achieved, the efficacy of this antigenic cocktail is quite high compared to other subunit protein vaccinations tried against experimental VL [79], [80] and needs further optimization. Finally, one clinical consequence of this work is that the protective antigen(s) might not be the only determinant of protection but also require highly immunopotentiating adjuvant to realize its full potential.

The development of a long-term protective immunity in terms of CD8^+^ T cells response is extremely important for successful vaccination against intracellular pathogens like *Leishmania*. In the recent past, many protein subunit vaccines that reported various levels of protection against *Leishmania*, often failed to generate sufficient long term memory against the disease [81]. Although, rHASPB1, rORFF, Leish-111f, and Leish-110f have reported sustained long term immunity against VL, they suffered either from using human incompatible adjuvant [82] or from low level of protection [83]. Importantly, Leish-111f and Leish-110f formulated with MPL-SE were human compatible, but did not show data for protection in both liver and spleen were not reported [82], [83]. Moreover, all these studies carried out in mice, challenged the vaccinated animals 3–4 weeks after the last boost. Our previous reports with crude leishmanial membrane antigen (LAg) [84] and native gp63 [85] entrapped in positively charged liposomes showed significant long term protection when challenged with virulent parasites 10–12 weeks after final immunization. However, one of the major drawbacks of these studies was the use of intraperitoneal route of immunization, not suitable for human use. This problem was overcome with the use of MPL-TDM adjuvant which could prime both CD8^+^ and CD4^+^ T cells when used in subcutaneous route with liposomal antigens: soluble leishmanial antigen (SLA) [86] and recombinant gp63 [11], leading to both short-term and long-term protection. The above results obtained so far and our preliminary survival analysis data in the present study do indicate an appreciable long-term protective response generated by the cocktail CPs formulated with MPL-TDM which was sustained at least up to 180 days post infection in hamsters. Unfortunately, mechanistic details of CD4^+^, CD8^+^ and regulatory T cells involved in vaccine mediated protection cannot be fully elucidated due to unavailability of hamster-specific reagents. This can be overcome with mice model in future which is the focus of our continuing research efforts.

## Supporting Information

Figure S1 **Phylogeny of** ***L. donovani*** **cysteine proteases.** A–C, Phylogram showing evolutionary relationship of cysteine protease A (A), B (B)and C(C) of different strains of *Leishmania* (*L. infantum*, *L. chagasi*, *L. mexicana*, *L. braziliensis*, *L. tropica*, *L. aethiopica*, *L. major* and *L. donovani*) with three different cathepsins in *Homo sapiens* at DNA level, using ClustalW Multiple Alignment in the FASTA format. The accession numbers of cysteine protease sequences used in the phylogenetic analysis are given in parentheses.
(TIF)

Figure S2 **Cloning of** ***cpa, cpb and cpc*** **from** ***L. donovani*** **.** A, lane 1–4, genomic DNA isolated from *L. donovani* (MHOM/IN/83/AG83) promastigotes. B, PCR amplification of *cpa, cpb and cpc* from *L. donovani* genomic DNA. C, cloning of *cpa* in pET28a vector. Lane 1, insert *cpa* (1.062 kb); lanes 2–5, NdeI/HindIII digested pET28a-*cpa* (vector size is ∼5 kb). D, cloning of *cpb* in pET28a vector Lane 1, insert *cpb* (1.335 kb); lane 2–5, NdeI/HindIII digested pET28a-*cpb* constructs. E, Cloning of *cpc* in pET28a vector. Lane 1, NdeI/HindIII digested pET28a-*cpc*; lane 2, insert *cpc* (1.038 kb); lane 3 and 4, PCR from positive clones pET28a-*cpc*.
(TIF)

Figure S3 **CLSM study of cellular localization of cationic liposomes in macrophage.** A, Elucidation of uptake mechanisms of Rh-123 (green) labeled cationic liposomes in hamster peritoneal MΦs treated with or without different biochemical inhibitors, studied by CLSM. Cell nuclei were stained with DAPI (blue). B, 3D confocal image showing colocalization of liposomes labeled with Rh-123 (green) with endosomes/lysosomes were labeled with LysoTracker Red (red) after 2 h of incubation with hamster peritoneal MΦs. White arrows indicate the occasions of coincidence (yellow: merge of red and green fluorescence) between the liposomes and endosome/lysosomes. Scale bars, 10 µm.
(TIF)

Figure S4 **Tapping mode AFM images of protein laden DSPC liposomes.** A, AFM images represented as two-dimensional graphics showing the clean spherical shaped liposomes encapsulating antigen (rCPC as reference protein). B, 3D image of the same liposomes. C, horizontal cross section indicating the height of the liposomes from the substratum.
(TIF)

Figure S5 **Protection against** ***L. donovani*** **in immunized hamsters.** A, Body weights before and at 2 and 3 months after challenge. Liver (B) and spleen (C) weight of immunized hamsters at designated time points after challenge. C, Upper panel shows representative image of spleens of different vaccinated groups at 3 months post infection.
(TIF)

Figure S6 **Hepatic histology sections stained with hematoxylin and eosin 2 months after challenge infection.** A, Liver architecture of normal and infected hamsters in comparison with immunized groups as indicated in at 2 months post-infection (upper panel magnification ×10; lower panel magnification ×40). B, Mature granuloma assembly (magnification ×100) in cocktail cysteine protease immunized animal. The results are representative of two independent experiments, for 3 individual hamsters per group.
(TIF)

Figure S7 **Evaluation of protection in hamsters at 3 months post infection.** A, DTH responses in free CP-immunized (without adjuvant) hamsters expressed as the differences (in millimeters) between the thicknesses of the test (antigen-injected) and control (PBS-injected) footpads. Results are shown as means ±S.E. for five animals per group and are representative of two independent experiments with similar results. B, Parasite burden (LDU) in the spleen at 3 months postinfection in hamsters immunized with CPA, CPB, CPC or cocktail with or without liposome adjuvant system. Data represent the mean ±S.E of five individual animals per group, representative of two independent experiments with similar results. P-values were assessed by Student's two-tail *t* test.
(TIF)

Materials and Methods S1 **Fixed-cell confocal laser scanning microscopy (CLSM).**
(DOC)

Table S1 **Primers used to amplify** ***cpa*** **,** ***cpb*** **and** ***cpc*** **from** ***L. donovani*** **(restriction sites underlined).**
(DOC)

Table S2 **Experimental design for vaccination.**
(DOC)

Table S3 **Sequence of forward and reverse primers used for quantitative real-time RT-PCR of cytokines from hamster.**
(DOC)
