# Correction: Combining Cationic Liposomal Delivery with MPL-TDM for Cysteine Protease Cocktail Vaccination against *Leishmania donovani*: Evidence for Antigen Synergy and Protection

**DOI:** 10.1371/journal.pntd.0004185

**Published:** 2015-10-20

**Authors:** Amrita Das, Nahid Ali

There are several errors within the text of this article.

In the *Cloning*, *expression and purification of L*. *donovani cysteine proteases a*, *b and c* subsection of the Materials and Methods section, the sixth sentence should read: The PCR amplified fragments were separately cloned into NdeI/BamHI or HindIII/BamHI sites of bacterial expression vector pET28a (Novagen, Madison, USA). The ninth sentence of the same section should read: For clone confirmation, approximately 1 μg plasmid DNA from an individual miniprep was double digested with the appropriate restriction enzymes (NdeI and BamHI for *cpa/b* or BamHI and HindIII for *cpc*) and the digest loaded onto a 1% agarose gel, in parallel with the molecular weight marker: 1 kb DNA ladder (Fermentas, USA).

The primer sequences for cloning *cpc* gene given in Table S1 should be as follows:

**Table:** 

*cpc* Forward	5′-CGG GAT CC ATG GCC CTC CGC GCC AAG TCT GCG CT -3′
*cpc* Reverse	5′- CCC AAG CTT CTA CTC CTG CGC GGG TGT GCC AGC AAC -3′
