# Supplementary figures and images for: Combining Cationic Liposomal Delivery with MPL-TDM for Cysteine Protease Cocktail Vaccination against Leishmania donovani : Evidence for Antigen Synergy and Protection
Source: PLoS Negl Trop Dis. 2014 Aug 21;8(8):e3091. doi: 10.1371/journal.pntd.0003091 (PMC4140747; doi:10.1371/journal.pntd.0003091)

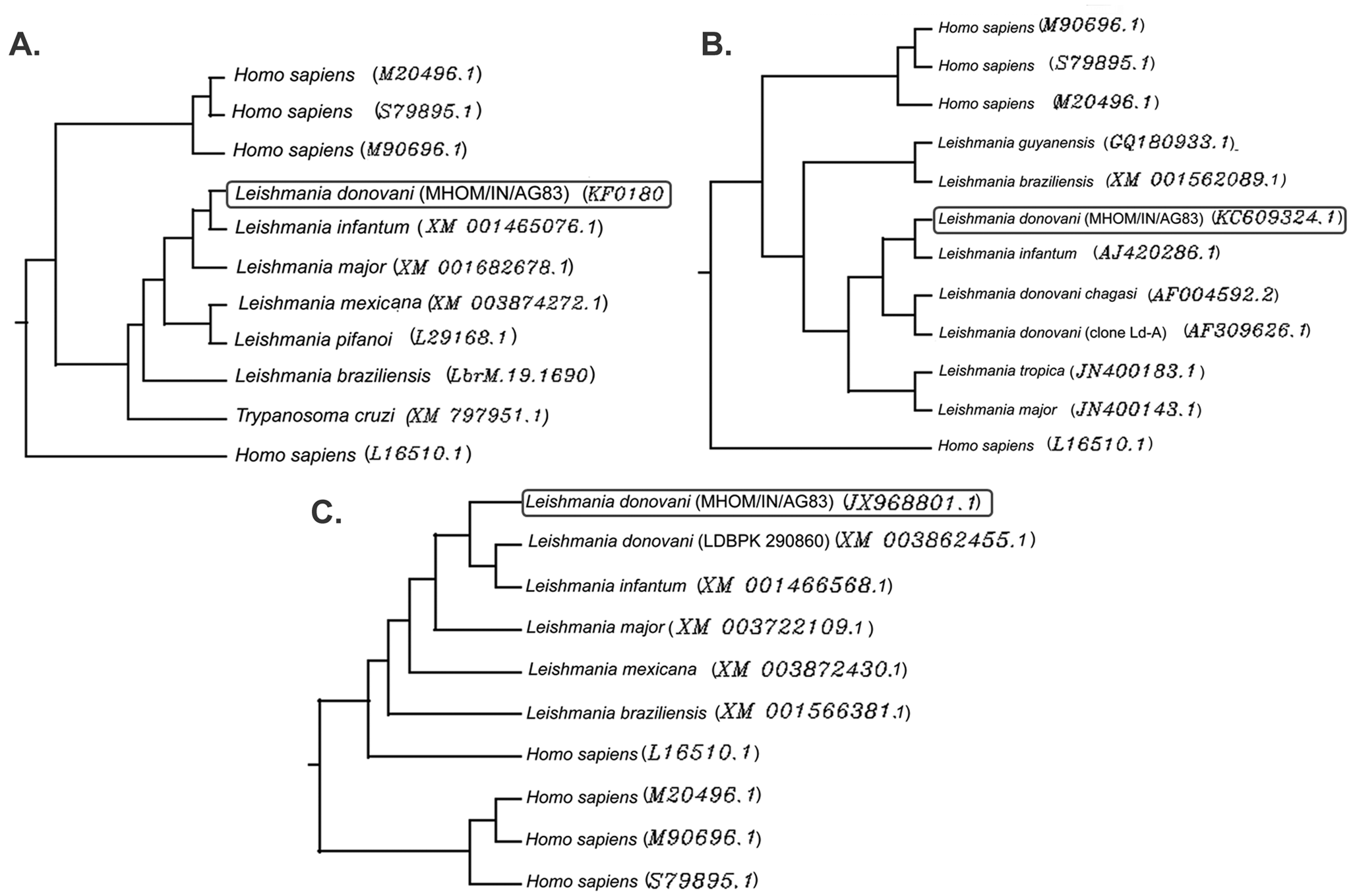

Supplement: Figure S1 — Phylogeny of L. donovani cysteine proteases. A–C, Phylogram showing evolutionary relationship of cysteine protease A (A), B (B)and C(C) of different strains of Leishmania (L. infantum, L. chagasi, L. mexicana, L. braziliensis, L. tropica, L. aethiopica, L. major and L. donovani) with three different cathepsins in Homo sapiens at DNA level, using ClustalW Multiple Alignment in the FASTA format. The accession numbers of cysteine protease sequences used in the phylogenetic analysis are given in parentheses. (TIF) [file pntd.0003091.s001.tif]

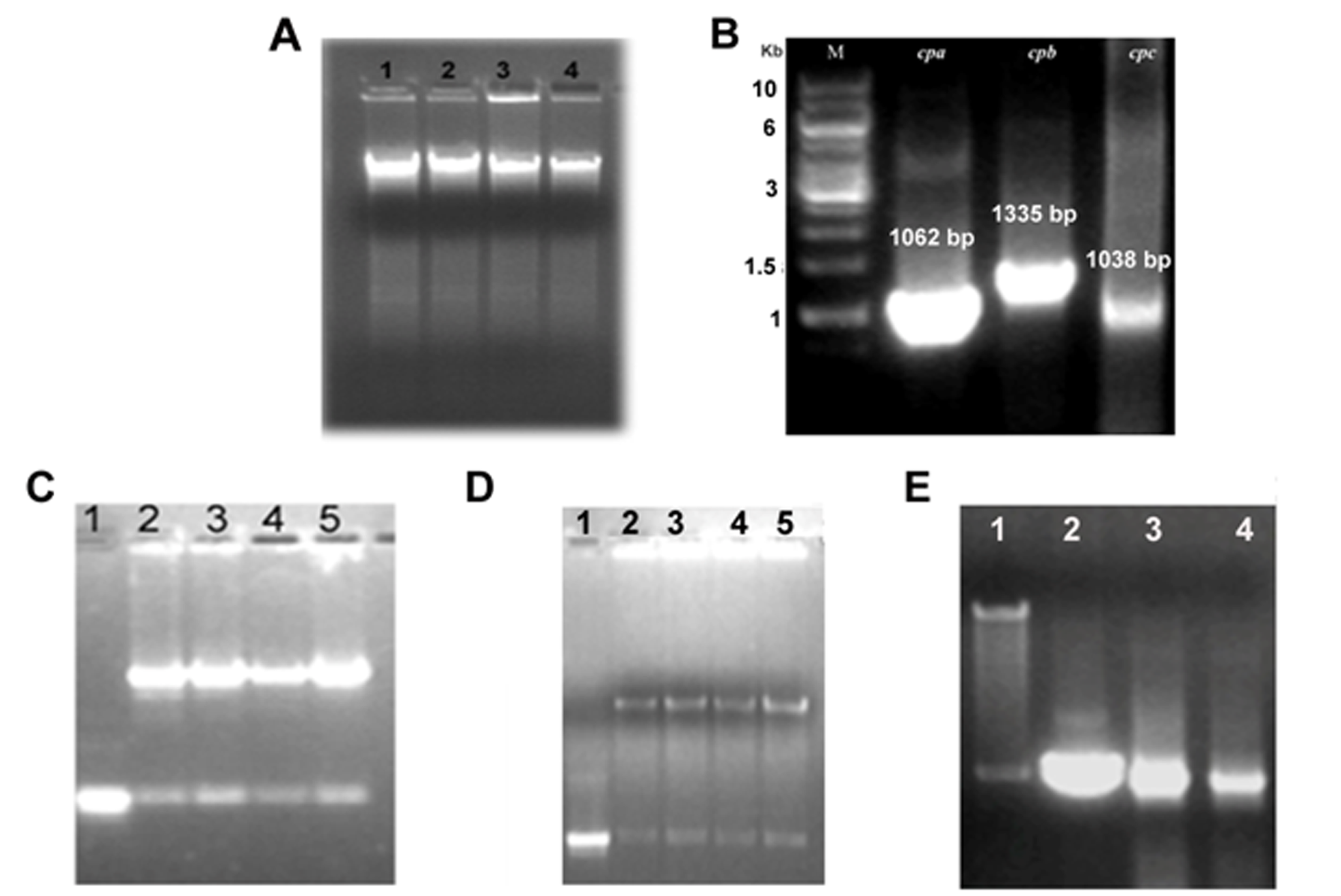

Supplement: Figure S2 — Cloning of cpa, cpb and cpc from L. donovani . A, lane 1–4, genomic DNA isolated from L. donovani (MHOM/IN/83/AG83) promastigotes. B, PCR amplification of cpa, cpb and cpc from L. donovani genomic DNA. C, cloning of cpa in pET28a vector. Lane 1, insert cpa (1.062 kb); lanes 2–5, NdeI/HindIII digested pET28a-cpa (vector size is ∼5 kb). D, cloning of cpb in pET28a vector Lane 1, insert cpb (1.335 kb); lane 2–5, NdeI/HindIII digested pET28a-cpb constructs. E, Cloning of cpc in pET28a vector. Lane 1, NdeI/HindIII digested pET28a-cpc; lane 2, insert cpc (1.038 kb); lane 3 and 4, PCR from positive clones pET28a-cpc. (TIF) [file pntd.0003091.s002.tif]

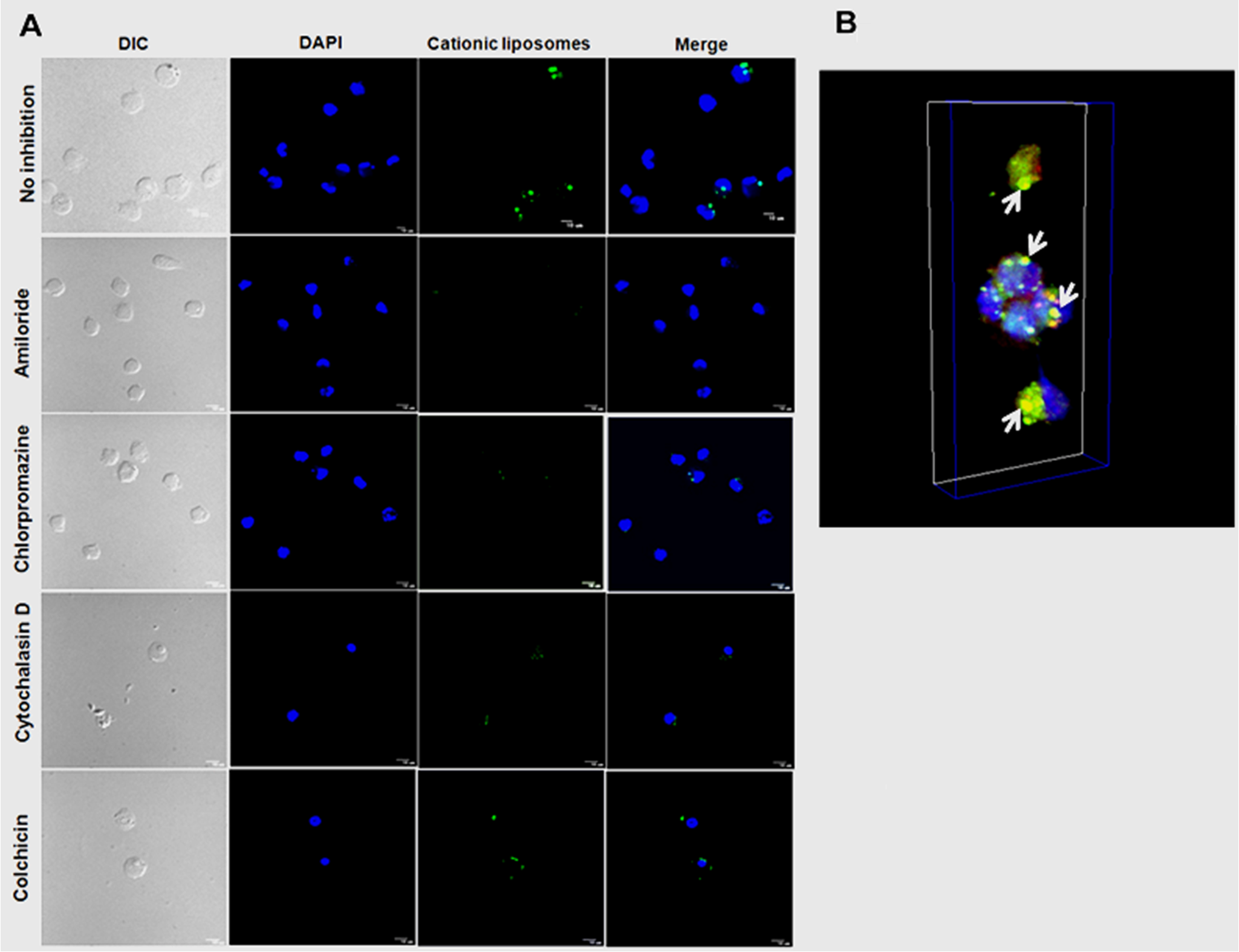

Supplement: Figure S3 — CLSM study of cellular localization of cationic liposomes in macrophage. A, Elucidation of uptake mechanisms of Rh-123 (green) labeled cationic liposomes in hamster peritoneal MΦs treated with or without different biochemical inhibitors, studied by CLSM. Cell nuclei were stained with DAPI (blue). B, 3D confocal image showing colocalization of liposomes labeled with Rh-123 (green) with endosomes/lysosomes were labeled with LysoTracker Red (red) after 2 h of incubation with hamster peritoneal MΦs. White arrows indicate the occasions of coincidence (yellow: merge of red and green fluorescence) between the liposomes and endosome/lysosomes. Scale bars, 10 µm. (TIF) [file pntd.0003091.s003.tif]

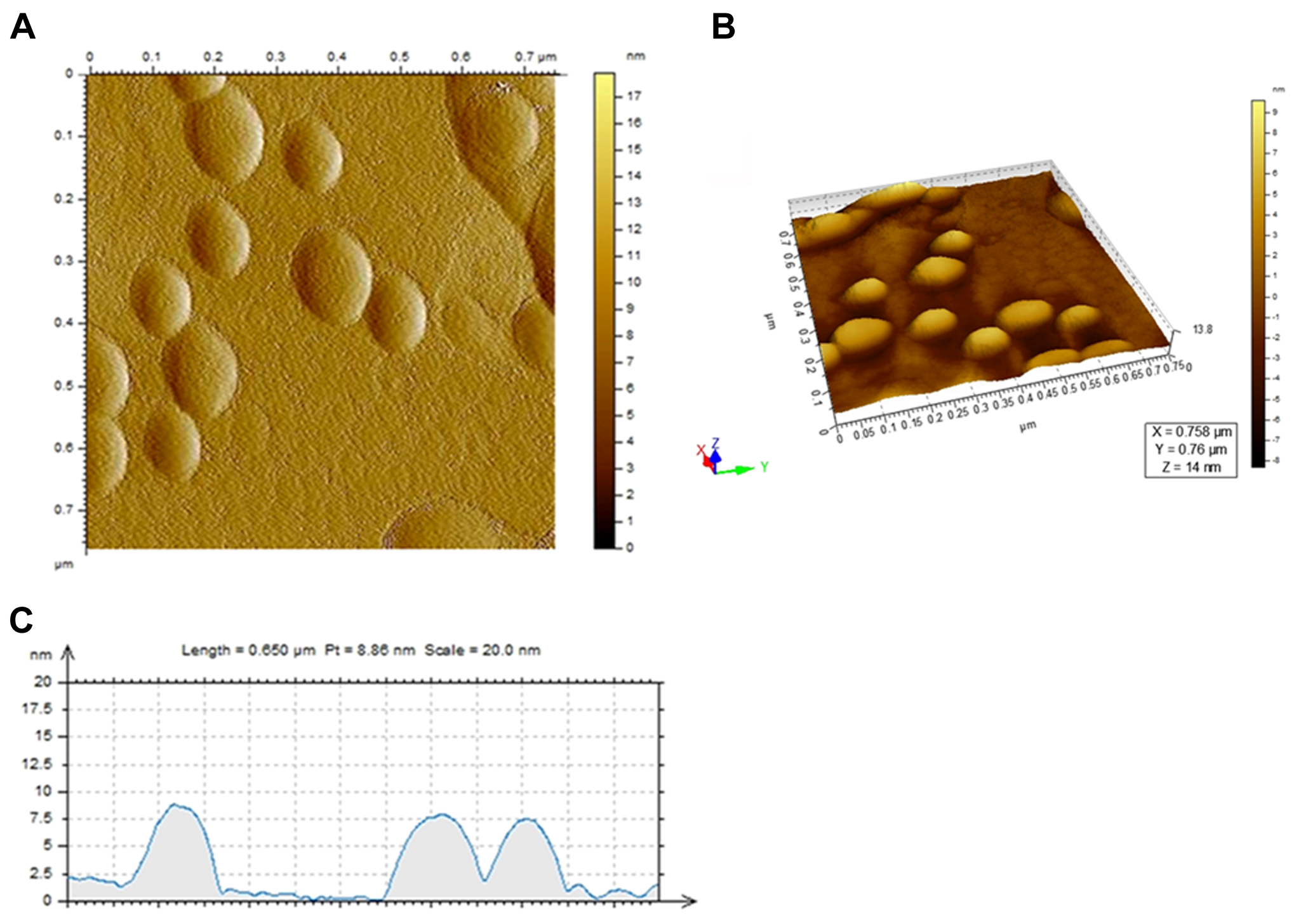

Supplement: Figure S4 — Tapping mode AFM images of protein laden DSPC liposomes. A, AFM images represented as two-dimensional graphics showing the clean spherical shaped liposomes encapsulating antigen (rCPC as reference protein). B, 3D image of the same liposomes. C, horizontal cross section indicating the height of the liposomes from the substratum. (TIF) [file pntd.0003091.s004.tif]

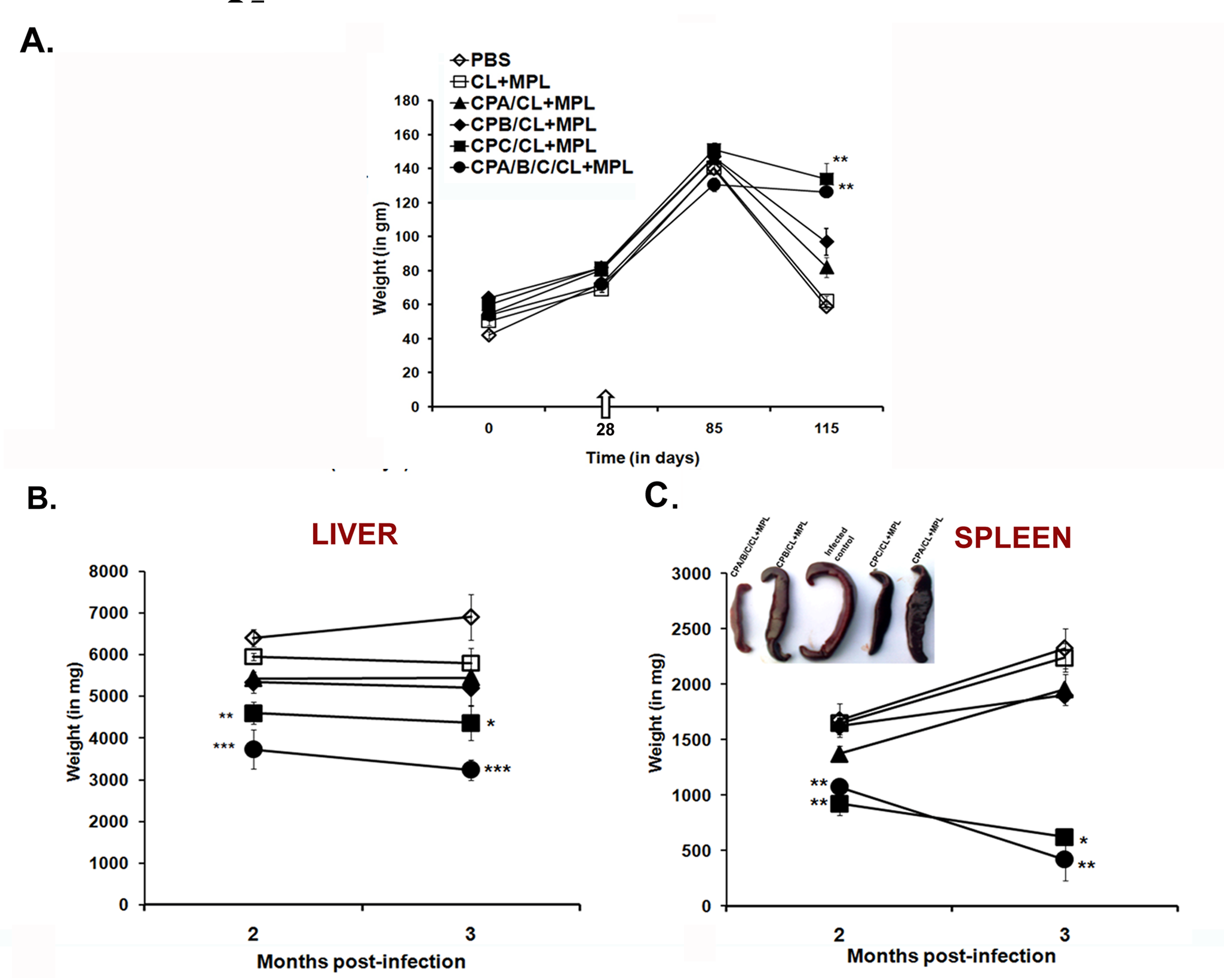

Supplement: Figure S5 — Protection against L. donovani in immunized hamsters. A, Body weights before and at 2 and 3 months after challenge. Liver (B) and spleen (C) weight of immunized hamsters at designated time points after challenge. C, Upper panel shows representative image of spleens of different vaccinated groups at 3 months post infection. (TIF) [file pntd.0003091.s005.tif]

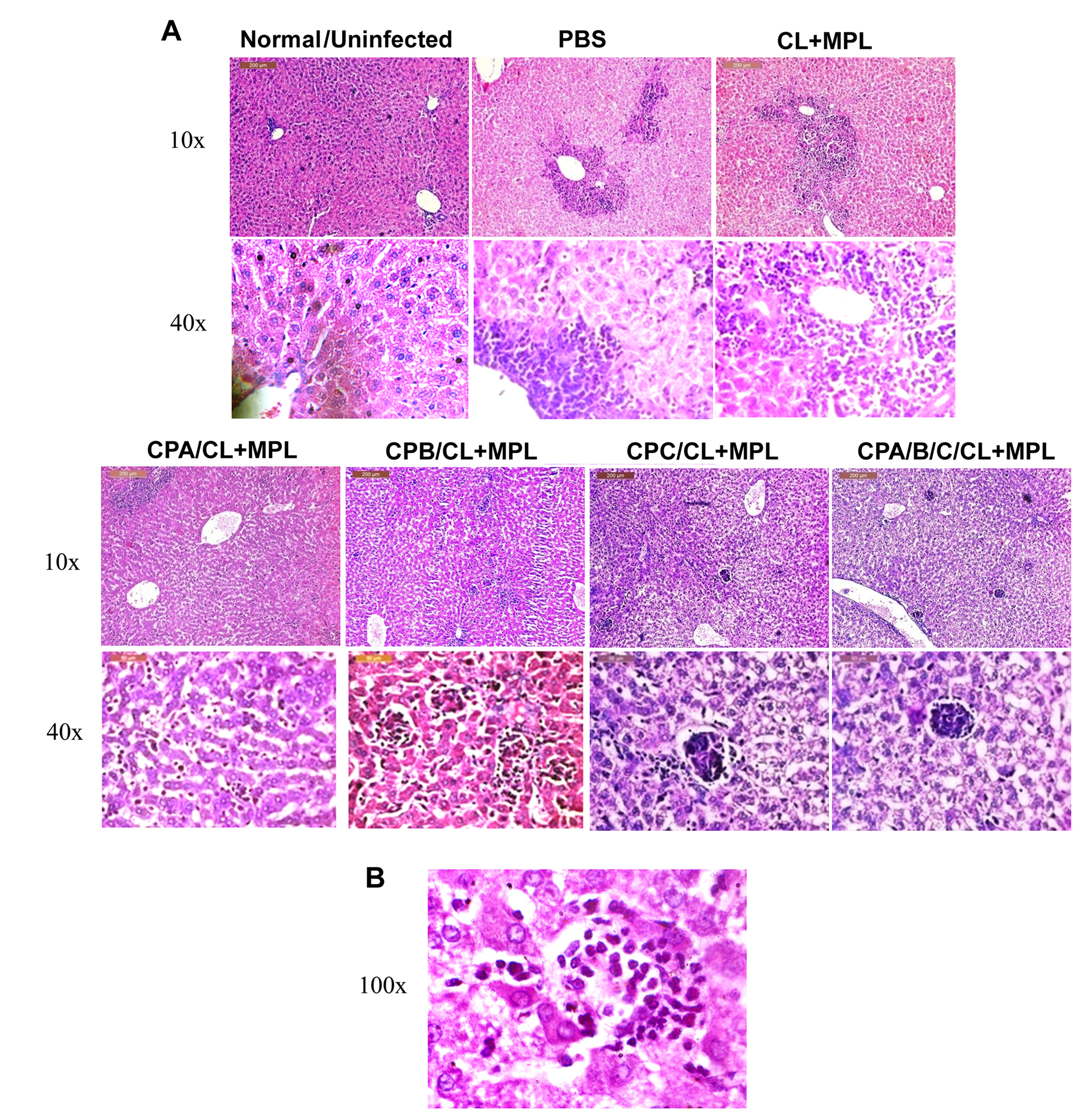

Supplement: Figure S6 — Hepatic histology sections stained with hematoxylin and eosin 2 months after challenge infection. A, Liver architecture of normal and infected hamsters in comparison with immunized groups as indicated in at 2 months post-infection (upper panel magnification ×10; lower panel magnification ×40). B, Mature granuloma assembly (magnification ×100) in cocktail cysteine protease immunized animal. The results are representative of two independent experiments, for 3 individual hamsters per group. (TIF) [file pntd.0003091.s006.tif]

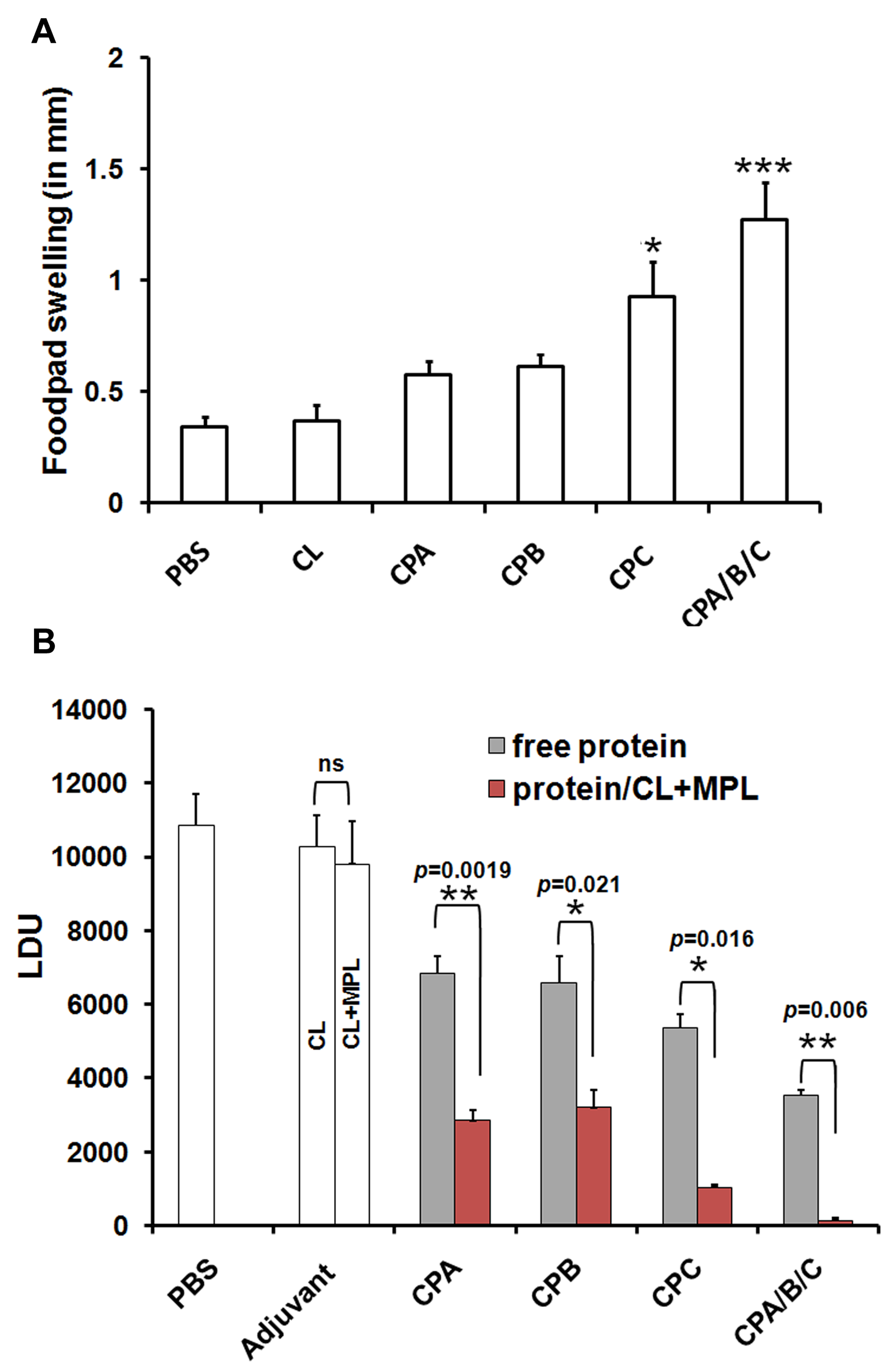

Supplement: Figure S7 — Evaluation of protection in hamsters at 3 months post infection. A, DTH responses in free CP-immunized (without adjuvant) hamsters expressed as the differences (in millimeters) between the thicknesses of the test (antigen-injected) and control (PBS-injected) footpads. Results are shown as means ±S.E. for five animals per group and are representative of two independent experiments with similar results. B, Parasite burden (LDU) in the spleen at 3 months postinfection in hamsters immunized with CPA, CPB, CPC or cocktail with or without liposome adjuvant system. Data represent the mean ±S.E of five individual animals per group, representative of two independent experiments with similar results. P-values were assessed by Student's two-tail t test. (TIF) [file pntd.0003091.s007.tif]
