# Supplementary material for: Combining Cationic Liposomal Delivery with MPL-TDM for Cysteine Protease Cocktail Vaccination against Leishmania donovani : Evidence for Antigen Synergy and Protection
Source: PLoS Negl Trop Dis. 2014 Aug 21;8(8):e3091. doi: 10.1371/journal.pntd.0003091 (PMC4140747; doi:10.1371/journal.pntd.0003091)
Supplement: Materials and Methods S1 — Fixed-cell confocal laser scanning microscopy (CLSM). (DOC) [file pntd.0003091.s008.doc]

**Supporting information**

***Table S1:*** *Primers used to amplify cpa, cpb and cpc from L. donovani (restriction sites underlined).*

| **Primer** | **Primer sequence** |
| --- | --- |
| *cpa* Forward | 5′- GGA ATT CCA TAT GGC GCG CCG CAA CCC CTT TTT GTT-3′ |
| *cpa* Reverse | 5′-CGG GAT CCG GCC GAA GAC GTC GGC ACGT-3′ |
| *cpb* Forward | 5′-GGA ATT CCA TAT GGAT CTC C CG GCC ATT TC T GCG CT-3′ |
| *cpb* Reverse | 5′-CGG GAT CC  CTC CTG CGC GGG TGT GCC AGC AAC-3′ |
| *cpc* Forward | 5′- GGA ATT CCA TAT GCCA GCG ACG TCA AGC GCC GCT-3′ |
| *cpc* Reverse | 5′-CGG GAT CC CTA CTC CTG CGC GTT TAT GCC AGC-3′ |

**Table S2:** Experimental design for vaccination.

| **Step** | **Day post immunization** | **Treatment** | **Dose** |
| --- | --- | --- | --- |
| **1** | 0 | Subcutaneous immunization of liposomal antigens with MPL-TDM. | 2.5µg of each antigen in liposomes with 25 µg of MPL-TDM |
| **2** | 14 | Booster of respective liposomal antigens along with MPL-TDM. | Similar to first immunization. |
| **3** | 21-22 | Assessment of DTH response | 50 µl of PBS or antigen (200 µg/ml) in control and test footpads respectively. |
| **4** | 24 | Collection of post-immunization sera | NA |
| **5** | 28 | Monitoring of body weight, antibody and DTH. Animals sacrificed for in vitro MΦ infection, cell proliferation, and cytokine analysis. Rest challenged with *L. donovani*. | Intracardiac challenge with 2.5×10^7^ freshly transformed promastigotes in 200 µl PBS. |
| **Step** | **Day post infection** | **Treatment** | **Dose** |
| **5** | 60 | Monitoring of body weight, DTH, antibody response. Animals sacrificed for cytokine analysis, evaluation of parasite burden, histology. | 50 µl of PBS or antigen (200µg/ml) in control and test footpads respectively for DTH. |
| **6** | 90 | Assessment of body weight, DTH, antibody response. Animals sacrificed for cytokine analysis, evaluation of parasite burden. | 50 µl of PBS or antigen (200µg/ml) in control and test footpads respectively for DTH. |

**Table S3:** Sequence of forward and reverse primers used for quantitative real-time RT-PCR of cytokines from hamster.

| **Primer** | **Primer sequence** |
| --- | --- |
| HGPRT forward | 5′-AGATCCACTCCCATAACTGTAGATTTTAT -3′ |
| HGPRT reverse | 5′- CATCCGCACCATTAATTTTTAAGTC -3′ |
| IFN-γ forward | 5′-GCTTAGATGTCGTGAATGG-3′ |
| IFN-γ reverse | 5′-GCTGCTGTTGAAGAAGTTAG-3′ |
| IL-4 forward | 5′- CCACGGAGAAAGACCTCATCTG -3′ |
| IL-4 reverse | 5′- GGGTCACCTCATGTTGGAAATAA -3′ |
| IL-12 forward | 5′-TATGTTGTAGAGGTGGACTG -3′ |
| IL-12 reverse | 5′-TTGTGGCAGGTGTATTGG -3′ |
| IL-10 forward | 5′- TGCCAAACCTTATCAGAAATG-3′ |
| IL-10 reverse | 5′- AGTTATCCTTCACCTGTTCC -3′ |
| IL-2 forward | 5′- AGTGCCTGGAAGAAGAA-3′ |
| IL-2 reverse | 5′- ATCTTCCAAGTGAAAGCTTTT-3′ |
| TNF-α forward | 5′- CGAGTGACAAGCCTGTAG-3′ |
| TNF-α reverse | 5′- TGATGGCAGAGAGGAGG-3′ |

**Materials and Methods S1**

**Fixed-cell confocal laser scanning microscopy (CLSM)**

To investigate the intracellular trafficking of liposomes, hamster peritoneal macrophages were incubated overnight on glass cover slips (22 mm^2^; 10^6^ macrophages per cover slip) in 0.5 ml of RPMI/10% FCS. After treatment with or without various inhibitors for designated time periods same as flowcytometry studies, liposomes labeled with Rh123 were added to 1 ml of the cell culture medium, followed by incubation for 1 h at 37°C. Cells were washed thrice with 0.02 M PBS and fixed with 4% paraformaldehyde (Sigma-Aldrich) for 10 minutes at room temperature. Fixed cells were finally washed with 0.02 M PBS and mounted onto glass slides with Prolong Gold containing DAPI (Invitrogen). Additionally, to track intracellular docking of labeled liposomes inside macrophages at 2 h, cells were incubated with LysoTracker™ Red (Molecular Probes, Eugene, Oregon). Fluorescence signals were viewed on an Andor Spinning Disk Live Cell Confocal microscope (Andor Technology) with a 60× oil-immersion objective and iQ Live Cell Imaging software. DAPI, Rh123 and LysoTracker™ Red were excited with a 405-nm, 488-nm and 577-nm argon laser, respectively. Adobe Photoshop (CS3, Adobe Systems) software was used to arrange the final images.

**Supporting information legends**

**Figure S1**. **Phylogeny of *L. donovani* cysteine proteases.** A-C, Phylogram showing evolutionary relationship of cysteine protease A (A), B (B)and C(C) of different strains of *Leishmania* (*L. infantum*, *L. chagasi*, *L. mexicana*, *L. braziliensis*, *L. tropica*, *L. aethiopica*, *L. major* and *L. donovani*) with three different cathepsins in *Homo sapiens* at DNA level, using ClustalW Multiple Alignment in the FASTA format. The accession numbers of cysteine protease sequences used in the phylogenetic analysis are given in parentheses.

**Figure S2**. **Cloning of *cpa, cpb and cpc* from *L. donovani*.** A, lane 1-4, genomic DNA isolated from *L. donovani* (MHOM/IN/83/AG83) promastigotes. B, PCR amplification of *cpa, cpb and cpc* from *L. donovani* genomic DNA. C, cloning of *cpa* in pET28a vector. Lane 1, insert *cpa* (1.062 kb); lanes 2-5, NdeI/HindIII digested pET28a-*cpa* (vector size is ~5kb). D, cloning of *cpb* in pET28a vector Lane 1, insert *cpb* (1.335 kb); lane 2-5, NdeI /HindIII digested pET28a-*cpb* constructs. E, Cloning of *cpc* in pET28a vector. Lane 1, NdeI /HindIII digested pET28a-*cpc*; lane 2, insert *cpc* (1.038 kb); lane 3 and 4, PCR from positive clones pET28a-*cpc*.

**Figure S3.** **CLSM study of cellular localization of cationic liposomes in macrophage.** A, Elucidation of uptake mechanisms of Rh-123 (green) labeled cationic liposomes in hamster peritoneal MΦs treated with or without different biochemical inhibitors, studied by CLSM. Cell nuclei were stained with DAPI (blue). B, 3D confocal image showing colocalization of liposomes labeled with Rh-123 (green) with endosomes/lysosomes were labeled with LysoTracker Red (red) after 2 h of incubation with hamster peritoneal MΦs. White arrows indicate the occasions of coincidence (yellow: merge of red and green fluorescence) between the liposomes and endosome/lysosomes. Scale bars, 10 μm.

**Figure S4. Tapping mode AFM images of protein laden DSPC liposomes.** A, AFM images represented as two-dimensional graphics showing the clean spherical shaped liposomes encapsulating antigen (rCPC as reference protein). B, 3D image of the same liposomes. C, horizontal cross section indicating the height of the liposomes from the substratum.

**Figure S5. Protection against *L. donovani* in immunized hamsters.** A, Body weights before and at 2 and 3 months after challenge. Liver (B) and spleen (C) weight of immunized hamsters at designated time points after challenge. C, Upper panel shows representative image of spleens of different vaccinated groups at 3 months post infection.

**Figure S6. Hepatic histology sections stained with hematoxylin and eosin 2 months after challenge infection.** A, Liver architecture of normal and infected hamsters in comparison with immunized groups as indicated in at 2 months post-infection (upper panel magnification x 10; lower panel magnification x 40). B, Mature granuloma assembly (magnification x 100) in cocktail cysteine protease immunized animal. The results are representative of two independent experiments, for 3 individual hamsters per group.

**Figure S7. Evaluation of protection in hamsters at 3 months post infection.** A, DTH responses in free CP-immunized (without adjuvant) hamsters expressed as the differences (in millimeters) between the thicknesses of the test (antigen-injected) and control (PBS-injected) footpads. Results are shown as means ±S.E. for five animals per group and are representative of two independent experiments with similar results. B, Parasite burden (LDU) in the spleen at 3 months post infection in hamsters immunized with CPA, CPB, CPC or cocktail with or without liposome adjuvant system. Data represent the mean ±S.E of five individual animals per group, representative of two independent experiments with similar results. P-values were assessed by Student’s two-tail *t* test.

**Table S1:** Primers used to amplify *cpa*, *cpb* and *cpc* from *L. donovani* (restriction sites underlined).

**Table S2:** Experimental design for vaccination.

**Table S3:** Sequence of forward and reverse primers used for quantitative real-time RT-PCR of cytokines from hamster.
