# Supplementary material for: Combining Cationic Liposomal Delivery with MPL-TDM for Cysteine Protease Cocktail Vaccination against Leishmania donovani : Evidence for Antigen Synergy and Protection
Source: PLoS Negl Trop Dis. 2014 Aug 21;8(8):e3091. doi: 10.1371/journal.pntd.0003091 (PMC4140747; doi:10.1371/journal.pntd.0003091)
Supplement: Table S1 — Primers used to amplify cpa , cpb and cpc from L. donovani (restriction sites underlined). (DOC) [file pntd.0003091.s009.doc]

***Table S1:*** *Primers used to amplify cpa, cpb and cpc from L. donovani (restriction sites underlined).*

| **Primer** | **Primer sequence** |
| --- | --- |
| *cpa* Forward | 5′- GGA ATT CCA TAT GGC GCG CCG CAA CCC CTT TTT GTT-3′ |
| *cpa* Reverse | 5′-CGG GAT CCG GCC GAA GAC GTC GGC ACGT-3′ |
| *cpb* Forward | 5′-GGA ATT CCA TAT GGAT CTC C CG GCC ATT TC T GCG CT-3′ |
| *cpb* Reverse | 5′-CGG GAT CC  CTC CTG CGC GGG TGT GCC AGC AAC-3′ |
| *cpc* Forward | 5′- GGA ATT CCA TAT GCCA GCG ACG TCA AGC GCC GCT-3′ |
| *cpc* Reverse | 5′-CGG GAT CC CTA CTC CTG CGC GTT TAT GCC AGC-3′ |
