# Supplementary material for: Combining Cationic Liposomal Delivery with MPL-TDM for Cysteine Protease Cocktail Vaccination against Leishmania donovani : Evidence for Antigen Synergy and Protection
Source: PLoS Negl Trop Dis. 2014 Aug 21;8(8):e3091. doi: 10.1371/journal.pntd.0003091 (PMC4140747; doi:10.1371/journal.pntd.0003091)
Supplement: Table S2 — Experimental design for vaccination. (DOC) [file pntd.0003091.s010.doc]

**Table S2:** Experimental design for vaccination.

| **Step** | **Day post immunization** | **Treatment** | **Dose** |
| --- | --- | --- | --- |
| **1** | 0 | Subcutaneous immunization of liposomal antigens with MPL-TDM. | 2.5µg of each antigen in liposomes with 25 µgof MPL-TDM |
| **2** | 14 | Booster of respective liposomal antigens along with MPL-TDM. | Similar to first immunization. |
| **3** | 21-22 | Assessment of DTH response | 50 µl of PBS or antigen (200 µg/ml) in control and test footpads respectively. |
| **4** | 24 | Collection of post-immunization sera | NA |
| **5** | 28 | Monitoring of body weight, antibody and DTH. Animals sacrificed for in vitro MΦ infection, cell proliferation, and cytokine analysis. Rest challenged with *L. donovani*. | Intracardiac challenge with 2.5×10^7^ freshly transformed promastigotes in 200 µl PBS. |
| **Step** | **Day post infection** | **Treatment** | **Dose** |
| **6** | 60 | Monitoring of body weight, DTH, antibody response. Animals sacrificed for cytokine analysis, evaluation of parasite burden, histology. | 50 µl of PBS or antigen (200µg/ml) in control and test footpads respectively for DTH. |
| **7** | 90 | Assessment of body weight, DTH, antibody response. Animals sacrificed for cytokine analysis, evaluation of parasite burden. | 50 µl of PBS or antigen (200µg/ml) in control and test footpads respectively for DTH. |
