# Supplementary material for: Combining Cationic Liposomal Delivery with MPL-TDM for Cysteine Protease Cocktail Vaccination against Leishmania donovani : Evidence for Antigen Synergy and Protection
Source: PLoS Negl Trop Dis. 2014 Aug 21;8(8):e3091. doi: 10.1371/journal.pntd.0003091 (PMC4140747; doi:10.1371/journal.pntd.0003091)
Supplement: Table S3 — Sequence of forward and reverse primers used for quantitative real-time RT-PCR of cytokines from hamster. (DOC) [file pntd.0003091.s011.doc]

**Table S3:** Sequence of forward and reverse primers used for quantitative real-time RT-PCR of cytokines from hamster.

| **Primer** | **Primer sequence** |
| --- | --- |
| HGPRT forward | 5′-AGATCCACTCCCATAACTGTAGATTTTAT -3′ |
| HGPRT reverse | 5′- CATCCGCACCATTAATTTTTAAGTC -3′ |
| IFN-γ forward | 5′-GCTTAGATGTCGTGAATGG-3′ |
| IFN-γ reverse | 5′-GCTGCTGTTGAAGAAGTTAG-3′ |
| IL-4 forward | 5′- CCACGGAGAAAGACCTCATCTG -3′ |
| IL-4 reverse | 5′- GGGTCACCTCATGTTGGAAATAA -3′ |
| IL-12 forward | 5′-TATGTTGTAGAGGTGGACTG -3′ |
| IL-12 reverse | 5′-TTGTGGCAGGTGTATTGG -3′ |
| IL-10 forward | 5′- TGCCAAACCTTATCAGAAATG-3′ |
| IL-10 reverse | 5′- AGTTATCCTTCACCTGTTCC -3′ |
| IL-2 forward | 5′- AGTGCCTGGAAGAAGAA-3′ |
| IL-2 reverse | 5′- ATCTTCCAAGTGAAAGCTTTT-3′ |
| TNF-α forward | 5′- CGAGTGACAAGCCTGTAG-3′ |
| TNF-α reverse | 5′- TGATGGCAGAGAGGAGG-3′ |
